# Power calculation for detecting interaction effect in cross-sectional stepped-wedge cluster randomized trials: an important tool for disparity research

**DOI:** 10.1186/s12874-024-02162-0

**Published:** 2024-03-02

**Authors:** Chen Yang, Asem Berkalieva, Madhu Mazumdar, Deukwoo Kwon

**Affiliations:** 1 Department of Population Health Science and Policy, Icahn School of Medicine at Mount Sinai, New York, NY USA; 2 Institute for Healthcare Delivery Science, Mount Sinai Health System, New York, NY USA; 3 Tisch Cancer Institute, Icahn School of Medicine at Mount Sinai, New York, NY USA; 4 Division of Clinical and Translational Sciences, Department of Internal Medicine, University of Texas Health Science Center at Houston, Houston, TX USA

**Keywords:** Cluster randomized trials, Stepped-wedge, Power calculation, Interaction effect, GEE

## Abstract

**Background:**

The stepped-wedge cluster randomized trial (SW-CRT) design has become popular in healthcare research. It is an appealing alternative to traditional cluster randomized trials (CRTs) since the burden of logistical issues and ethical problems can be reduced. Several approaches for sample size determination for the overall treatment effect in the SW-CRT have been proposed. However, in certain situations we are interested in examining the heterogeneity in treatment effect (HTE) between groups instead. This is equivalent to testing the interaction effect. An important example includes the aim to reduce racial disparities through healthcare delivery interventions, where the focus is the interaction between the intervention and race. Sample size determination and power calculation for detecting an interaction effect between the intervention status variable and a key covariate in the SW-CRT study has not been proposed yet for binary outcomes.

**Methods:**

We utilize the generalized estimating equation (GEE) method for detecting the heterogeneity in treatment effect (HTE). The variance of the estimated interaction effect is approximated based on the GEE method for the marginal models. The power is calculated based on the two-sided Wald test. The Kauermann and Carroll (KC) and the Mancl and DeRouen (MD) methods along with GEE (GEE-KC and GEE-MD) are considered as bias-correction methods.

**Results:**

Among three approaches, GEE has the largest simulated power and GEE-MD has the smallest simulated power. Given cluster size of 120, GEE has over 80% statistical power. When we have a balanced binary covariate (50%), simulated power increases compared to an unbalanced binary covariate (30%). With intermediate effect size of HTE, only cluster sizes of 100 and 120 have more than 80% power using GEE for both correlation structures. With large effect size of HTE, when cluster size is at least 60, all three approaches have more than 80% power. When we compare an increase in cluster size and increase in the number of clusters based on simulated power, the latter has a slight gain in power. When the cluster size changes from 20 to 40 with 20 clusters, power increases from 53.1% to 82.1% for GEE; 50.6% to 79.7% for GEE-KC; and 48.1% to 77.1% for GEE-MD. When the number of clusters changes from 20 to 40 with cluster size of 20, power increases from 53.1% to 82.1% for GEE; 50.6% to 81% for GEE-KC; and 48.1% to 79.8% for GEE-MD.

**Conclusions:**

We propose three approaches for cluster size determination given the number of clusters for detecting the interaction effect in SW-CRT. GEE and GEE-KC have reasonable operating characteristics for both intermediate and large effect size of HTE.

**Supplementary Information:**

The online version contains supplementary material available at 10.1186/s12874-024-02162-0.

## Background

The push towards studying and improving racial and ethnic disparities in healthcare has grown in recent years. Across various settings, retrospective studies continue to demonstrate that Black patients are less likely to obtain adequate care and outcomes than that of White patients [1–4]. Within the field of palliative care, Black patients are less likely to receive advanced care planning and to enroll in palliative care than other racial groups [5–7]. One step towards mitigating these findings includes implementing interventions, first introduced through randomized controlled trials, which work to close the health disparity gap between minority and non-minority patients. Examples include properly training and removing racial bias among physicians [6], improving communication between physicians and patients [8, 9], and integrating automated tools into physicians’ decision making to prompt care [10]. While the interventions should work to increase quality of care among all patients, in order to truly tackle the issue of disparity, they should have a greater impact on minority patients, as their baseline care is usually less adequate than that of non-minorities. In the statistical models analyzing the impact of these interventions, the main interest is the magnitude and significance of the interaction term between race and the exposure variable.

Interventions aimed at reducing racial disparities in healthcare often tackle change at the provider or clinic level, suggesting the use of cluster randomized trials (CRT). This has been conducted in several published trials. In a two-arm, parallel group CRT, researchers aimed to increase unmet palliative care needs among both Black and White intensive care unit (ICU) families by introducing an automated web app, aimed at improving communication between physicians and families [9]. While this study relied on a parallel group CRT, these designs are not always feasible, particularly in cases where the intervention cannot be denied to half of the participants. In these situations, the stepped-wedge cluster randomized trial (SW-CRT) is an appealing design [11]. Appealing features of SW-CRTs include having each cluster act as its own control and not needing to withhold the intervention from any patient due to the key feature of only needing unidirectional cross-over of clusters from control to intervention in a time-staggered manner. However, from a statistical perspective, the SW-CRT design gives us complex correlation structures compared to the standard CRT design due to the uni-directional crossover from control to intervention.

Several approaches for optimal sample size determination of SW-CRT for estimating overall treatment effect (OTE) are available in published literature [12–17] for both continuous and binary outcomes. Various software implementations of these approaches are summarized in existing literature [18, 19]. In the study design stage for CRT, detecting the overall intervention effect is the main consideration in the sample size determination/power calculation, and examination of differential effect by group is the secondary objective [9, 20]. However, in trials assessing health disparities, the key interest is in examining whether the intervention effect differs among study subgroups. In healthcare delivery research, examination of differential effect by group would be also considered in the study planning stage. Sample size calculation methods for continuous outcomes based on the interaction level have been studied extensively in recent advances [21, 22]. However, conducting power calculations around the interaction level in these types of trials is challenging, and sample size determination for detecting an interaction effect between intervention status variable and a key covariate in the SW-CRT study has not been proposed yet. Since binary outcome measures are the most common outcome in SW-CRTs for healthcare delivery research, we focus on binary outcomes in this study.

There is limited evidence of similar health-equity focused CRTs properly powering their studies for the interaction term or providing enough transparency of methodologies used. The parallel-group CRT assessing palliative care needs among ICU families powered their study around the OTE for all patients as well as by independently powering each racial subgroup for identical OTEs. However, the study admitted they likely did not have enough power for the interaction effect between the exposure and race [9]. Similarly, a SW-CRT aimed at increasing the rates of advanced care planning (ACP) among African-American patients through a structured ACP intervention conducted their main sample size calculation around the OTE, and they briefly mentioned they had sufficient power in the secondary objective for detecting a reduction in the racial disparity gap [8].Limited information was provided on how this calculation was conducted, preventing future researchers from utilizing their design. Aside from the statistical drawbacks that come with under-powering a study, failing to address that the effect size for the minority group should be larger than that of the non-minority group is an issue. By designing the study to achieve the same effect size among all subgroups, the issue of the disparity gap will remain. Instead, these types of studies should be powered to detect a larger effect size in the minority group.

Researchers at Mount Sinai Hospital System (MSHS) are planning to conduct a cross-sectional SW-CRT for advanced cancer patients who are at high risk for dying within a short period of time (less than 6 months). There would be two choices for the patients and their family members that oncologists are expected to discuss as the ‘goals of care’ (GoC): (1) remaining in active treatment with curative intent and (2) moving into hospice care for management of pain and discomfort only. A machine-learning based predictive model for 6-month predictive mortality has already been optimized with retrospective data and validated with prospective data. A clinical decision support system (CDSS) with information from the predictive model will guide the physician through the electronic medical record (EMR) system to have a GoC for the patient predicted at high-risk for mortality. Physicians in the intervention arm will also be trained on how to have the GoC with patients and family members and will be shown data on low GoC conversations for the minority (Black and Hispanic) patients at MSHS. Therefore, the intervention of the planned SW-CRT is a combination of training and a CDSS based alert about prediction. The physicians in the control arm will have neither the CDSS alert nor the training. The binary outcome in this case is having the GoC conversation or not. The binary covariate is race (White/Black). It is hypothesized that the proportion of GoC in Black patients will increase at a higher rate than for White patients with the same intervention. This is equivalent to testing the hypothesis that the interaction effect between intervention and race is significant.

In this article, our main focus of the study design is on the cross-sectional SW-CRT design, in which the outcome is measured on individuals within each cluster at each time period. For example, in a cross-sectional SW-CRT to evaluate a hospital-based psychological care quality improvement intervention for cancer patients, the binary outcome of health-related quality of life improvement (improved vs worse/unchanged) was measured among cross-sections of individuals attending the hospitals during each time-period [6]. We propose approaches to this problem in a cross-sectional SW-CRT setting with an individual binary covariate. We utilize the generalized estimating equation (GEE) model setting for these formulations for binary outcomes. We organize this article in the following manner. In Sect. 2, we introduce the three proposed approaches for obtaining the optimal sample size for detecting interaction effect in the cross-sectional SW-CRT. In Sect. 3, we describe the planned simulation study for estimating comparative empirical type I and type II error rates for several settings. In Sect. 4, we show simulation results. Section 5 discusses a motivational example for this manuscript. We provide an illustration of the proposed approaches using a real data example from the MSHS study. We conclude our article in Sect. 6 and Sect. 7 with some discussion and plans for expanding our study to continuous and censored endpoints as well as detecting the interaction effect with cluster-level covariates.

## Methods

### Statistical approaches in the cross-sectional SW-CRT

In general, the design and analysis for SW-CRT has been based on either conditional model approaches or a marginal model approach. Conditional model approaches require specification of fixed effects and random effects. Linear mixed models (LMM) are used for continuous outcomes, while generalized linear mixed models (GLMM) are used for binary outcomes. When we consider a simpler model without an interaction between intervention and covariates, fixed effects include period effect terms and intervention effect term, while random effects include random cluster effect and cluster-by-time interaction. For the closed cohort SW-CRT, the random effect for the repeated measures from an individual within the same cluster is needed. Using these random effect terms, conditional approaches enable us to have flexible random effect structures. While the LMM benefits from the flexible random effect structures due to the interpretation that the intervention effect is unrelated to these random effect structures. The interpretation of the intervention effect is not straightforward for the GLMM since it depends on the specification of the random effect structure. For sample size determination, flexible modeling of random effects requires distributional assumptions. These assumptions need more information in the study design stage. However, the marginal model approach provides intuitively straightforward interpretation of intervention effect since it focuses on the population-averaged intervention effect. This approach needs two specifications: marginal mean model and a working correlation structure. Unlike conditional model approaches, the intervention effect interpretation does not depend on correlation structure modeling [23].

### Models for estimating the heterogeneity of effect in the cross-sectional SW-CRT

We consider the marginal model via the generalized estimating equation (GEE) approach for the cross-sectional SW-CRT design, where we focus on the interaction effect between the intervention and a binary individual covariate. Let ${Y}_{ijk}$ be a binary outcome of the $k$ th individual ($k\in \left\{1,...,{m}_{i}\right\}$) in the $i$ th cluster ($i\in \left\{1,...,I\right\}$) at the $j$ th time interval ($j\in \left\{1,...,J\right\}$), where *m*_*i*_ denotes cluster size for cluster *i*, *I* denotes the total number of clusters, and *J* denotes total time steps and ${Y}_{ijk}=1$ denotes the event of interest and ${Y}_{ijk}=0$ otherwise. A GEE model is formulated for the cross-sectional SW-CRT as follows:

1 $${\text{logit}}\left({\mu }_{ijk}\right)={\theta }_{0}+{\gamma }_{j}+{\theta }_{1}{W}_{ij}+{\theta }_{2}{X}_{ijk}+{\theta }_{3}{W}_{ij}{X}_{ijk}$$

where ${\mu }_{ijk}={\mathbb{E}}\left[{Y}_{ijk}\right]$, denotes the marginal mean response of ${Y}_{ijk}$, ${\theta }_{0}$ is the baseline log-odds of the outcome in the control group corresponding to the reference group for the binary covariate (${X}_{ijk}\in \left\{\mathrm{0,1}\right\}$, ${X}_{ijk}=0$ represents the reference group and ${X}_{ijk}=1$ represents the other), and ${\gamma }_{j}$ is the period fixed effect for the $j$ th time interval. ${W}_{ij}$ is the design indicator; ${W}_{ij}=1$ means that all individuals in cluster $i$ at time interval $j$ receive the intervention and ${W}_{ij}=0$ otherwise. ${\theta }_{1}$ is the overall treatment effect (OTE), ${\theta }_{2}$ is main effect of binary covariate, and ${\theta }_{3}$ captures the interaction between treatment and the binary covariate (i.e., heterogeneity in treatment effect denoted by HTE). For identification, we set ${\gamma }_{1}=0$. We use $\Theta =({\gamma }_{2},\cdots ,{\gamma }_{J},{\theta }_{0},{\theta }_{1},{\theta }_{2},{\theta }_{3})\in {\mathbb{R}}^{J+3}$ for the parameters and variance of ${Y}_{ijk}$ is defined as ${\upsilon }_{ijk}$, where ${\mathbb{R}}^{d}$ represents the space of all $d$-dimensional real vectors and ${\upsilon }_{ijk}={\mu }_{ijk}(1-{\mu }_{ijk})$ for binary outcome. Hence model (1) can be written as

$${\text{logit}}\left({\mu }_{ijk}\right)={\mathbf{M}}_{ijk}^{\intercal }\Theta$$

where $${\mathbf{M}}_{ijk}:={\left[\begin{array}{ccccc}1& {\mathbf{e}}_{j}^{\intercal }& {W}_{ij}& {X}_{ijk}& {W}_{ij}{X}_{ijk}\end{array}\right]}^{\intercal }\in {\mathbb{R}}^{J+3}$$ with ${\mathbf{e}}_{j}$ as the vector of length $J-1$ with all elements equal to 0 except the $j-1$ th element, which is equal to 1. Now by stacking ${\mathbf{M}}_{ijk}$’s as the matrix of $J{m}_{i}$ rows and $J+3$ columns, namely,

$${\mathbf{M}}_{i}={\left[\begin{array}{ccccc}{\mathbf{M}}_{i11}& \cdots & {\mathbf{M}}_{i1{m}_{i}}& \cdots & {\mathbf{M}}_{iJ{m}_{i}}\end{array}\right]}^{\intercal }\in {\mathbb{R}}^{J{m}_{i}\times \left(J+3\right)}$$

We reach

$${\text{logit}}\left({\mathbf{u}}_{i}\right)={\mathbf{M}}_{i}\Theta \in {\mathbb{R}}^{J{m}_{i}}$$

where $${\mathbf{u}}_{i}={\left[\begin{array}{cccccc}{\mu }_{i11}& \cdots & {\mu }_{i1{m}_{i}}& {\mu }_{i21}& \cdots & {\mu }_{iJ{m}_{j}}\end{array}\right]}^{\intercal }$$ and the function logit here is applied to the vector ${\mathbf{u}}_{i}$ elementwise.

If the HTE is to be tested with respect to ${X}_{ijk}$, the interaction effect parameter ${\theta }_{3}$, instead of the OTE ${\theta }_{1}$, should be considered for the sample size calculation. In this case, the null hypothesis ${H}_{0}: {\theta }_{3}=0$ is to be tested against an alternative hypothesis ${H}_{{\text{a}}}: {\theta }_{3}=\delta$ for some prespecified HTE level $\delta \ne 0$. For the purpose of simplification, we assume the SW-CRT has equal cluster sizes, i.e. ${m}_{1}=\cdots ={m}_{I}=m$. If the HTE level ${\theta }_{3}$ is estimated by a consistent, asymptotically normally distributed estimator ${\widehat{\theta }}_{3,m}$, then the power is approximately calculated using the two-tailed Wald test,

2 $${\text{power}}=\Phi \left(\frac{\delta }{\sqrt{{\mathbb{V}}ar\left({\widehat{\theta }}_{3,m}\right)}}-{z}_{1-\alpha /2}\right)$$

where $\Phi$ is the standard normal distribution function and ${z}_{1-\alpha /2}$ is the ${(1-\alpha /2)}^{{\text{th}}}$ standard normal quantile. When estimating the OTE, t distribution is also recommended particularly for SW-CRT designs with small number of clusters:

3 $${\text{power}}= {\Phi }_{t,{\text{df}}}\left(\frac{\delta }{\sqrt{{\mathbb{V}}ar\left({\widehat{\theta }}_{3,m}\right)}}-{t}_{1-\alpha /2, {\text{df}}}\right)$$

where ${\Phi }_{t,{\text{df}}}$ is the cumulative t distribution function with certain degree of freedom (df). Although the proposed method can be used to determine either the number of clusters or cluster size, in this article we focus on determining cluster size and we provide R code for both methods. The use of formula (2) or (3) requires an approximation of ${\mathbb{V}}ar\left({\widehat{\theta }}_{3,m}\right)$, which is the (J + 3, J + 3)^th^ element in the model-based variance–covariance matrix, ${\mathbb{V}}ar\left({\widehat{\Theta }}_{m}\right)$ where ${\widehat{\Theta }}_{m}$ is the GEE estimator of $\Theta$. In this article, we provide the sandwich form of approximation for ${\mathbb{V}}ar\left({\widehat{\Theta }}_{m}\right)$’s, which arises from the GEE method. Due to the presence of the individual binary covariate, the closed form of the sample size calculation is not available. We consider two correlation structures: a simple exchangeable correlation structure and a nested exchangeable correlation structure which can be based on the values of within-period correlation and between-period correlation. The intraclass correlation (ICC) measures the correlation on the outcome for different individuals in the same cluster within a given time period. The within-period correlation is same as the intraclass correlation (ICC). The cluster autocorrelation (CAC) is the correlation between the population means from the same cluster at two different time periods and is defined as the ratio of between-period correlation and within-period correlation. Hence, a simple exchangeable correlation structure has the same value for within-period correlation and between-period correlation (i.e., CAC = 1), and a nested correlation structure has different values for within-period correlation and between-period correlation. In general, between-period correlation is smaller than within-period correlation (0 < CAC < 1).

Now denote the ICC as $\alpha \in \left(-\mathrm{1,1}\right)$ such that ${\mathbb{C}}ov\left({Y}_{ijk},{Y}_{ijk{\prime}}\right)=\alpha \sqrt{{\upsilon }_{ijk}{\upsilon }_{ijk{\prime}}}$ for $k{\prime}\ne k$ and the CAC as $\rho \in \left[-\mathrm{1,1}\right]$ such that ${\mathbb{C}}ov\left({Y}_{ijk},{Y}_{ij{\prime}k{\prime}}\right)=\alpha \rho \sqrt{{\upsilon }_{ij{\prime}k}{\upsilon }_{ijk{\prime}}}$ for $j{\prime}\ne j$. Then the variance–covariance matrix of the binary outcomes is ${\mathbf{V}}_{i}={\mathbb{V}}ar\left({\mathbf{Y}}_{i}\right)={\mathbf{A}}_{i}^\frac{1}{2}{\mathbf{R}}_{i}\left(\alpha ,\rho \right){\mathbf{A}}_{i}^\frac{1}{2}$ for cluster $i$, where

$${\mathbf{R}}_{i}\left(\alpha ,\rho \right)=\alpha \rho {\mathbf{J}}_{J{m}_{i}}+\alpha \left(1-\rho \right){\mathbf{I}}_{J}\otimes {\mathbf{J}}_{{m}_{i}}+\left(1-\alpha \right){\mathbf{I}}_{J{m}_{i}}$$

${\mathbf{I}}_{n}$ is the $n\times n$ identity matrix, ${\mathbf{J}}_{n}$ is an $n\times n$ matrix with all elements equal to 1, and ${\mathbf{A}}_{i}={\text{diag}}\left({\upsilon }_{i11},\cdots ,{\upsilon }_{i1{m}_{i}},\cdots {\upsilon }_{iJ1},\cdots ,{\upsilon }_{iJ{m}_{i}}\right)$. Hence the GEE is

4 $${\text{U}}\left(\Theta \right)=\sum_{i=1}^{I}\frac{\partial {\mathbf{u}}_{i}}{\partial\Theta }{\mathbf{V}}_{i}^{-1}\left({\mathbf{Y}}_{i}-{\mathbf{u}}_{i}\right)=0$$

By solving the GEE (3), the resulting ${\widehat{\Theta }}_{m}$ satisfies

$${\widehat{\Theta }}_{m}-\Theta ={\left(\sum_{i=1}^{I}\frac{\partial {\mathbf{u}}_{i}}{\partial\Theta }{\mathbf{V}}_{i}^{-1}\frac{\partial {\mathbf{u}}_{i}}{\partial\Theta }\right)}^{-1}\sum_{i=1}^{I}\frac{\partial {\mathbf{u}}_{i}}{\partial\Theta }{\mathbf{V}}_{i}^{-1}\left({\mathbf{Y}}_{i}-{\mathbf{u}}_{i}\right)+{O}_{\mathbb{P}}\left({I}^{-1}\right)$$

By ignoring the ${O}_{\mathbb{P}}\left({I}^{-1}\right)$ term if $I$ is sufficiently large, we have

5 $${\mathbb{V}}ar\left({\widehat{\Theta }}_{m}\right)={\mathbb{V}}ar\left({\widehat{\Theta }}_{m}-\Theta \right)\approx {\left(\sum_{i=1}^{I}\frac{\partial {\mathbf{u}}_{i}}{\partial\Theta }{\mathbf{V}}_{i}^{-1}\frac{\partial {\mathbf{u}}_{i}}{\partial\Theta }\right)}^{-1}={\left(\sum_{i=1}^{I}{\mathbf{M}}_{i}^{\intercal }{\mathbf{A}}_{i}{\mathbf{V}}_{i}^{-1}{\mathbf{A}}_{i}{\mathbf{M}}_{i}\right)}^{-1}$$

As a result, ${\mathbb{V}}ar\left({\widehat{\Theta }}_{m}\right)$ can be approximately calculated as

6 $${\mathbb{V}}ar\left({\widehat{\Theta }}_{m}\right)\approx {\left(\sum_{i=1}^{I}{\mathbf{M}}_{i}^{\intercal }{{\varvec{\Sigma}}}_{i}^{-1}{\mathbf{M}}_{i}\right)}^{-1}$$

where ${{\varvec{\Sigma}}}_{i}={\mathbf{A}}_{i}^{-\frac{1}{2}}{\mathbf{R}}_{i}\left(\alpha ,\rho \right){\mathbf{A}}_{i}^{-\frac{1}{2}}$ for cluster $i$. Equation (6) leads to a model-based (naïve) variance estimator.

7 $$\widehat{{\mathbb{V}}ar}\left({\widehat{\Theta }}_{m}\right)={\left(\sum_{i=1}^{I}{\mathbf{M}}_{i}^{\intercal }{\widehat{{\varvec{\Sigma}}}}_{i}^{-1}{\mathbf{M}}_{i}\right)}^{-1}$$

where ${\widehat{{\varvec{\Sigma}}}}_{i}={\mathbf{A}}_{i}^{-\frac{1}{2}}{\mathbf{R}}_{i}\left(\widehat{\alpha },\widehat{\rho }\right){\mathbf{A}}_{i}^{-\frac{1}{2}}$ with $\widehat{\alpha }$ and $\widehat{\rho }$ also obtained by solving the GEE (4). Note that for power calculation we do not have data. Instead, we have assumptions about the values of $\alpha$ and $\rho$. Hence we may compute $\widetilde{{\mathbb{V}}ar}\left({\widehat{\Theta }}_{m}\right)$ without any data analysis, i.e. $\widetilde{{\mathbb{V}}ar}\left({\widehat{\Theta }}_{m}\right)$ is a deterministic quantity rather than a random variable. We call power calculation method (2) or (3) based on the $\widetilde{{\mathbb{V}}ar}\left({\widehat{\Theta }}_{m}\right)$ with specific values of $\alpha$ and $\rho$ “GEE” for simplicity.

### Bias-correction sandwich variance approaches

It is well-known that $\widetilde{{\mathbb{V}}ar}\left({\widehat{\theta }}_{3,m}\right)$, the $\left(J+3,J+3\right)$ th element of $\widetilde{{\mathbb{V}}ar}\left({\widehat{\Theta }}_{m}\right)$, is less than the true value of ${\mathbb{V}}ar\left({\widehat{\theta }}_{3,m}\right)$ due to ignoring the ${O}_{\mathbb{P}}\left({I}^{-1}\right)$ term particularly for cases with small $I$. From the perspective of data analysis, if the number of clusters $I$ is small, bias-correction techniques are recommended for obtaining $\widehat{{\mathbb{V}}ar}\left({\widehat{\Theta }}_{m}\right)$ to mitigate increased risk of type I errors [24]. In this article, we consider two bias-correction techniques from data analysis to adjust $\widetilde{{\mathbb{V}}ar}\left({\widehat{\theta }}_{3,m}\right)$ for small number of clusters: 1) the Kauermann and Carroll (KC) [25] Correction; and 2) the Mancl and DeRouen (MD) [26] Correction. Both bias-correction techniques lead to the following modified form of approximation for ${\mathbb{V}}ar\left({\widehat{\Theta }}_{m}\right)$:

8 $${\mathbb{V}}ar\left({\widehat{\Theta }}_{m}\right)\approx {\left(\sum_{i=1}^{I}{\mathbf{M}}_{i}^{\intercal }{{\varvec{\Sigma}}}_{i}^{-1}{\mathbf{M}}_{i}\right)}^{-1}\left(\sum_{i=1}^{I}{\mathbf{M}}_{i}^{\intercal }{{\varvec{\Omega}}}_{i}^{-1}{\mathbf{M}}_{i}\right){\left(\sum_{i=1}^{I}{\mathbf{M}}_{i}^{\intercal }{{\varvec{\Sigma}}}_{i}^{-1}{\mathbf{M}}_{i}\right)}^{-1}$$

where ${{\varvec{\Omega}}}_{i}$ is the corresponding residual-modified version of ${{\varvec{\Sigma}}}_{i}$ with the form

$${{\varvec{\Omega}}}_{i}={\left({{\varvec{\Sigma}}}_{i}^{-1}{\mathbf{A}}_{i}^{-1}{\mathbf{F}}_{i}{\mathbf{A}}_{i}{{\varvec{\Sigma}}}_{i}{\mathbf{A}}_{i}{\mathbf{F}}_{i}^{\intercal }{\mathbf{A}}_{i}^{-1}{{\varvec{\Sigma}}}_{i}^{-1}\right)}^{-1}$$

where the residuals modification matrices ${\mathbf{F}}_{i}$ is defined as one of

$${\mathbf{F}}_{i}^{{\text{KC}}}={\left({\mathbf{I}}_{J{m}_{i}}-{\mathbf{H}}_{i}\right)}^{-\frac{1}{2}}\in {\mathbb{R}}^{J{m}_{i}\times J{m}_{i}}$$

and

$${\mathbf{F}}_{i}^{{\text{MD}}}={\left({\mathbf{I}}_{J{m}_{i}}-{\mathbf{H}}_{i}\right)}^{-1}\in {\mathbb{R}}^{J{m}_{i}\times J{m}_{i}}$$

depending on the selection of bias-correction technique (KC or MD). The matrix ${\mathbf{H}}_{i}$ is defined as

$${\mathbf{H}}_{i}={\mathbf{A}}_{i}{\mathbf{M}}_{i}{\left(\sum_{i=1}^{I}{\mathbf{M}}_{i}^{\intercal }{{\varvec{\Sigma}}}_{i}^{-1}{\mathbf{M}}_{i}\right)}^{-1}{\mathbf{M}}_{i}^{\intercal }{{\varvec{\Sigma}}}_{i}^{-1}{\mathbf{A}}_{i}^{-1}\in {\mathbb{R}}^{J{m}_{i}\times J{m}_{i}}.$$

Note that by writing

$${\mathbf{F}}_{i}={\left({\mathbf{I}}_{J{m}_{i}}-{\mathbf{H}}_{i}\right)}^{0}={\mathbf{I}}_{J{m}_{i}}$$

the right hand-side of (8) collapses to $\widetilde{{\mathbb{V}}ar}\left({\widehat{\Theta }}_{m}\right)$, which means $\widetilde{{\mathbb{V}}ar}\left({\widehat{\Theta }}_{m}\right)$ is also a particular case of the right hand-side of (8) like the KC and MD cases. For this reason, we may distinguish our power calculation methods by the choice of ${\mathbf{F}}_{i}$ in the right hand-side of (8), namely, “GEE” refers to the method with ${\mathbf{F}}_{i}={\mathbf{I}}_{J{m}_{i}}$; “GEE-KC” refers to the method with ${\mathbf{F}}_{i}={\mathbf{F}}_{i}^{{\text{KC}}}$; and “GEE-MD” refers to the method with ${\mathbf{F}}_{i}={\mathbf{F}}_{i}^{{\text{MD}}}$. Again, we do not estimate ${\mathbb{V}}ar\left({\widehat{\Theta }}_{m}\right)$ because ${\mathbf{I}}_{J{m}_{i}}$, ${\mathbf{F}}_{i}^{{\text{KC}}}$, and ${\mathbf{F}}_{i}^{{\text{MD}}}$ are known given the assumption of ${\mathbf{R}}_{i}\left(\alpha ,\rho \right)$ ‘s and the parameters therein at the design stage.. In other words, GEE-KC and GEE-MD are proposed to improve the predicted power based on GEE in the situation that number of clusters is small.

### Simulation study

We performed a simulation study to examine the operating characteristics of the three proposed methods described in Sect. 2 to detect the interaction effect between intervention and binary individual covariate in cross-sectional SW-CRT. The simulation study was conducted using statistical software R version 4.2.2.

### Scheme for generating simulation datasets

We simulate binary outcomes ${Y}_{ijk}$’s based on marginal means ${\mathbf{u}}_{i}$ and correlation matrix ${\mathbf{R}}_{i}\left(\alpha ,\rho \right)$. Note that we have

$${\mathbb{E}}\left[{\mathbf{Y}}_{i}{\mathbf{Y}}_{i}^{\intercal }\right]={\mathbf{A}}_{i}^\frac{1}{2}{\mathbf{R}}_{i}\left(\alpha ,\rho \right){\mathbf{A}}_{i}^\frac{1}{2}+{\mathbf{u}}_{i}{\mathbf{u}}_{i}^{\intercal }$$

where $${\mathbf{Y}}_{i}={\left[\begin{array}{cccccc}{Y}_{i11}& \cdots & {Y}_{i1m}& {Y}_{i21}& \cdots & {Y}_{iJm}\end{array}\right]}^{\intercal }.$$

To simulate ${\mathbf{Y}}_{i}$, we consider the copula-based method for multivariate binary outcomes [27, 28] which assumes ${Y}_{ijk}=1\left\{{Z}_{ijk}\le {\Phi }^{-1}\left({\mu }_{ijk}\right)\right\}$, for $j=1,\dots ,J$ and $k=1,\dots ,m$, where $1\left\{\cdot \right\}$ is the indicator function, $\Phi$ is the standard normal CDF and the random vector, $${\mathbf{Z}}_{i}={\left[\begin{array}{cccccc}{Z}_{i11}& \cdots & {Z}_{i1m}& {Z}_{i21}& \cdots & {Z}_{iJm}\end{array}\right]}^{\intercal }\sim {N}_{Jm}\left(0,{{\varvec{\Xi}}}_{i}\right)$$ for some correlation matrix ${{\varvec{\Xi}}}_{i}\in {\mathbb{R}}^{Jm\times Jm}$ (hence ${Z}_{ijk}\sim N\left(\mathrm{0,1}\right)$ for all $j=1,\dots ,J$ and $k=1,\dots ,m$ marginally). The elements of ${{\varvec{\Xi}}}_{i}$ corresponding to the location of ${Z}_{ijk}$ and ${Z}_{ij\mathrm{^{\prime}}k\mathrm{^{\prime}}}$ within ${\mathbf{Z}}_{i}$ are defined as

$${\xi }_{ijk,ij\mathrm{^{\prime}}k\mathrm{^{\prime}}}=Corr\left({Z}_{ijk},{Z}_{ij\mathrm{^{\prime}}k\mathrm{^{\prime}}}\right)={\mathbb{E}}\left[{Z}_{ijk}{Z}_{ij\mathrm{^{\prime}}k\mathrm{^{\prime}}}\right]$$

For all $j,{j}{\prime}=1,\dots ,J$ and $k,{k}{\prime}=1,\dots ,m$. To ensure that ${\mathbf{R}}_{i}\left(\alpha ,\rho \right)$ is the correlation matrix of ${\mathbf{Y}}_{i}$, ${\xi }_{ijk,ij\mathrm{^{\prime}}k\mathrm{^{\prime}}}$ is determined by solving the equation

$${C}_{{\xi }_{ijk,ij\mathrm{^{\prime}}k\mathrm{^{\prime}}}}\left({\mu }_{ijk},{\mu }_{ij\mathrm{^{\prime}}k\mathrm{^{\prime}}}\right)={\mathbb{E}}\left[{Y}_{ijk}{Y}_{ij\mathrm{^{\prime}}k\mathrm{^{\prime}}}\right]$$

where ${C}_{{\xi }_{ijk,ij\mathrm{^{\prime}}k\mathrm{^{\prime}}}}$ is a bivariate Gaussian copula with correlation parameter ${\xi }_{ijk,ij\mathrm{^{\prime}}k\mathrm{^{\prime}}}$. We compute the bivariate copula components using the “pbinormcop” function of the R package “VGAM”.

Note that the resulting ${{\varvec{\Xi}}}_{i}$ is not always positively definite [29]. In this case, we need to modify this matrix to force it to become non-negative definite such that we may simulate the random vector ${\mathbf{Z}}_{i}$ using the modified ${{\varvec{\Xi}}}_{i}$. An eigenvalue modification trick [30] is employed for this purpose. Values for parameters for the simulation study setups are shown in Table 1. We set ${\theta }_{0}={\text{log}}(0.15/0.85)$ representing 15% prevalence of outcome for control group with reference group of binary covariate. This prevalence rate came from our motivational example. We included increasing secular time trend by specifying ${\gamma }_{2}=0.1,{\gamma }_{3}=0.2,{\gamma }_{4}=0.3,$ and ${\gamma }_{5}=0.4$. In power calculation the secular time trend fixed effects should be determined either from existing literature or by a preliminary analysis. In the Supplementary materials of (16), a demonstration of the ‘power.ap’ function in the R package CRTpowerdist considers categorical time trend using coefficients 1, 2, 3, and 4 for Gaussian outcomes; for binary outcomes using logit link these values are too large for computation. As a result, we use 0.1, 0.2, 0.3, and 0.4 instead for demonstration purpose only. We simulated binary covariate, ${X}_{ijk}$ using the cluster prevalence levels 30% and 50%, respectively. These two values also came from our motivational example in which 30% of patients are Black and 48% of patients are female. We plan to evaluate the statistical operating characteristics of HTE when we have imbalanced and balanced binary covariates. For OTE, we set ${\theta }_{1}={\text{log}}\left(1.35\right)$ and ${\theta }_{1}={\text{log}}\left(1.68\right)$ representing a small and intermediate effect sizes for the intervention effect and ${\theta }_{2}={\text{log}}\left(1.5\right)$ for the binary covariate. The main quantity of interest is HTE, ${\theta }_{3}$. We considered two values, ${\text{log}}\left(1.5\right)$ and ${\text{log}}\left(2\right)$, to achieve an intermediate and large effect size, respectively.

**Table 1:** Setup for simulation study for parameter values except interaction along with the number of clusters, time points, and cluster size

Value for parameter	Reason for determining value
${\theta }_{0}={\text{log}}\left(0.15/0.85\right)$	the true log odds ratio of the outcome in the control group (${W}_{ijk}=0$) with reference group for binary covariate (${X}_{ijk}=0$) for k^th^ individual in i^th^ cluster at j^th^ period. We assume that prevalence of outcome is 15%
${\gamma }_{1}=0, {\gamma }_{2}=0.1,{\gamma }_{3}=0.2,{\gamma }_{4}=0.3,{\gamma }_{5}=0.4$	Increasing secular trend
${{\theta }_{1}={\text{log}}\left(1.35\right); \theta }_{1}={\text{log}}\left(1.68\right)$	Chosen arbitrarily for small and intermediate effect size for intervention effect
30%, 50%	Prevalence rate for binary covariate
${\theta }_{2}={\text{log}}\left(1.5\right)$	Chosen arbitrarily for intermediate effect size for binary covariate
${\theta }_{3}={\text{log}}\left(1.5\right); {\theta }_{3}={\text{log}}\left(2\right)$	Chosen arbitrarily for intermediate and large effect size for interaction
$I=8, 20, 40$	Number of clusters
$J=5$	Number of time steps
$m=\mathrm{20,40}, 60, \mathrm{80,120}$	Cluster size
$ICC=0.1$ $ICC=0.1$;$CAC= 0.8$	For a simple exchangeable correlation structure For a nested exchangeable correlation structure

### SW-CRT design related parameters

Relatively small number of clusters are often used in cross-sectional SW-CRT. A literature review found that 50% of 56 cross-sectional SW-CRTs reviewed had fewer than 10 clusters [31]. Therefore, we simulated eight clusters (I = 8), five time periods (J = 5), and several fixed cluster sizes (20, 40, 60, 80, and 120). Hence, the total numbers of subjects in this simulation study were 800, 1,600, 2,400, 3,200, and 4,800 individuals, respectively.

Since we used GEE models, we needed to specify working correlation matrix structure. Although there are three distinct correlation structures that are typically used in the cross-sectional SW-CRT: simple exchangeable correlation structure, nested exchangeable correlation structure, and exponential decay correlation structure, we decided to simulate the first two because they are most commonly used in simulation studies [17, 32, 33]. For both correlation structures used, we set ICC to be 0.1. We considered a CAC value of 0.8 for the nested exchangeable correlation structure.

### Measure of operating characteristic for simulation study

In the simulation study we examined simulated power and empirical type I error rate for three proposed approaches from 1,000 generated datasets using the simulation setup shown in Table 1.

For the null scenario with ${\theta }_{3}=0$, the empirical Type I error rate (${\psi }_{0}$) was calculated as the proportion of false rejections among the 1,000 tests for ${\theta }_{3}$; for the nonnull scenarios (${\theta }_{3}={\text{log}}\left(1.5\right)$ and ${\text{log}}\left(2\right)$), the simulated power (${\varphi }_{0}$) was calculated as the proportion of correct rejections among the 1,000 tests for ${\theta }_{3}$. When fitting simulated samples using GEE, the naive standard error estimation is considered because modifications KC and MD are added in the approximation of ${\mathbb{V}}ar\left({\widehat{\Theta }}_{m}\right)$.

### Comparison between simulated power and predicted power for sample size determination

We conduct a simulation study to examine sample size determination, provided in terms of cluster size, from each approach given the numbers of clusters and periods. We consider two distinct values for the prevalence of binary covariate (30% and 50%), the OTE effect size (${\text{log}}\left(1.35\right)$ and ${\text{log}}\left(1.68\right)$), and the HTE effect size (${\text{log}}\left(1.5\right)$ and ${\text{log}}\left(2\right)$).

The predicted powers/Type I errors are obtained by our proposed methods (GEE, GEE-KC and GEE-MD) using Eq. (2) given the design parameters. The simulated powers/empirical Type I errors are obtained by simulation based on the same set of design parameters. While the simulated powers are usually seen as the true powers for the given design due to the law of large numbers, they might be very time-consuming depending on the complexity of the simulation scheme and the true value of OTE/HTE. For this reason, we expect to have priori information of a suitable range of cluster sizes such that our simulated power may achieve the target power (80% for example) without conducting simulation studies. To this end, mathematical approximation methods are employed to compute the predicted powers/Type I errors as fast feedbacks to guessing values of cluster sizes and to help determine the suitable cluster sizes.

### Sensitivity analysis

In the sensitivity analysis for the three proposed approaches using different numbers of clusters and cluster sizes on simulated power, we consider two additional numbers of clusters (I = 20 and 40) and five additional cluster sizes (m = 20, 40, 60, 80, and 100) given a prevalence rate of 50% for the binary covariate and an intermediate OTE effect size (${\theta }_{1}={\text{log}}\left(1.68\right)$). Once again, we consider intermediate and large effect sizes for HTE, the quantity of interest. From different combinations of the number of clusters and cluster sizes, we examine whether an increase in the number of clusters improves statistical power compared to an increase in cluster size indirectly. We also examine the tradeoff between the number of clusters and cluster size in simulated power. We consider two total number of observations per step (160 and 800) and then two combinations of the number of clusters and cluster sizes for a given total number of observations. One pair represents small number of clusters and relatively bigger cluster size and another pair represents larger number of clusters and small cluster size. Hence, I = 8 & m = 20 and I = 40 & m = 4 for 160 observations per step and I = 8 & m = 100 and I = 40 & m = 20 for 800 observations per step. We also examine the impact of ICC and CAC on simulated power for the above setup. We consider three setups for ICC and CAC: ICC = 0.1 & CAC = 1; ICC = 0.1 & CAC = 0.8; and ICC = 0.05 & CAC = 0.4.

## Results

### Comparison of sample size determination

In Tables 2 and 3, we evaluate the difference between predicted standard error (SE) and empSE defined in Table 10 of [34] ($\Delta$ SE), simulated power (${\varphi }_{0}$) and empirical Type I error (${\psi }_{0}$) based on the naïve and robust SEs of GEE for a determined cluster size satisfying 80% power from our three approaches using the following values (I = 8, J = 5, ${\theta }_{0}={\text{log}}\left(0.15/0.85\right)$). We evaluate this for a simple exchangeable correlation structure (ICC = 0.1) and for a nested exchangeable correlation structure (ICC = 0.1, CAC = 0.8). We also consider two different values for effect sizes of OTE and HTE as well as the prevalence rate of the binary covariate. The cluster sizes are taken as multiples of ten intentionally to generate integer number of observations for both X = 0 and X = 1 within each cluster. For the same reason, only even numbers are taken into account under the scenario of prevalence = 50%. For all cases, empirical Type I errors fall into the 95% confidence interval of nominal significance level (5%). For both a simple exchangeable correlation structure and a nested exchangeable correlation structure, simulated powers of GEE-KC and GEE-MD are above the upper limit of the 95% confidence interval of the predicted power ($\varphi$) for both intermediate and large effect sizes of HTE. GEE maintains the simulated power inside of the 95% confidence interval of the predicted power in both correlation structures when naïve SE is used. If robust SE is used, simulated powers of GEE are also above the upper limit of the 95% confidence interval of the predicted power ($\varphi$) for both intermediate and large effect sizes. When we calculate the difference between simulated power (${\varphi }_{0}$) and predicted power ($\varphi$), GEE shows the smallest among the three approaches. This comes from relatively bigger sample size determination for the two bias-correction approaches provided.

**Table 2:** Required cluster sizes to achieve 80% predicted power to detect interaction effect sizes for a simple exchangeable correlation structure with the number of clusters = 8 and ICC = 0.1

${\theta }_{1}$(OTE)	${\theta }_{3}$(HTE)	Method	Prevalence rate for binary individual covariate, X													
			30%							50%						
			Cluster size	$\Delta$ SE	Naïve SE		Robust SE		$\varphi$	Cluster size	$\Delta$ SE	Naïve SE		Robust SE		$\varphi$
					${\psi }_{0}$	${\varphi }_{0}$	${\psi }_{0}$	${\varphi }_{0}$				${\psi }_{0}$	${\varphi }_{0}$	${\psi }_{0}$	${\varphi }_{0}$	
${\text{log}}\left(1.35\right)$	${\text{log}}\left(1.5\right)$	GEE	110	-0.005	0.046	0.789	0.136	0.825	0.801	98	-0.005	0.038	0.785	0.118	0.824	0.803
		Mean(GEE,GEE-KC)	120	-	0.054	0.820	0.119	**0.859**	0.801	108	-	0.042	0.829	0.129	**0.859**	0.804
		GEE-KC	130	0.007	0.053	**0.864**	0.109	**0.897**	0.801	116	0.007	0.052	**0.867**	0.119	**0.879**	0.804
		GEE-MD	160	0.019	0.040	**0.918**	0.109	**0.916**	0.815	138	0.019	0.048	**0.923**	0.121	**0.916**	0.805
	${\text{log}}\left(2\right)$	GEE	40	-0.015	0.059	0.819	0.121	**0.852**	0.827	34	0.001	0.061	0.797	0.122	0.833	0.810
		GEE-KC	50	0.004	0.049	**0.883**	0.107	**0.906**	0.847	40	0.020	0.044	**0.856**	0.130	**0.896**	0.809
		GEE-MD	60	0.025	0.055	**0.950**	0.113	**0.951**	0.851	48	0.042	0.048	**0.891**	0.144	**0.929**	0.813
${\text{log}}\left(1.68\right)$	${\text{log}}\left(1.5\right)$	GEE	110	-0.002	0.046	0.791	0.118	0.822	0.807	96	-0.009	0.054	0.799	0.129	**0.841**	0.804
		Mean(GEE,GEE-KC)	120	-	0.055	0.829	0.126	**0.860**	0.807	106	-	0.046	0.820	0.116	**0.847**	0.805
		GEE-KC	130	0.009	0.052	**0.847**	0.121	**0.867**	0.806	114	0.002	0.048	**0.833**	0.115	**0.869**	0.805
		GEE-MD	160	0.022	0.039	**0.908**	0.119	**0.922**	0.819	134	0.015	0.053	**0.891**	0.109	**0.904**	0.801
	${\text{log}}\left(2\right)$	GEE	40	-0.005	0.053	0.811	0.124	0.846	0.831	34	-0.002	0.046	**0.773**	0.110	0.821	0.818
		GEE-KC	50	0.013	0.041	**0.889**	0.116	**0.905**	0.850	40	0.017	0.036	**0.857**	0.112	**0.890**	0.816
		GEE-MD	60	0.034	0.063	**0.929**	0.128	**0.949**	0.854	46	0.039	0.050	**0.907**	0.147	**0.927**	0.804

Estimated required cluster size was determined to achieve at least 80% predicted power. Bias of the standard error $\Delta$ SE, empirical Type I error ${\psi }_{0}$, simulated power ${\varphi }_{0}$ (GEE fitted using both naïve and robust SE, respectively), and predicted power $\varphi$ were obtained from the GEE power calculator with ICC = 0.1 using three methods GEE, GEE-KC, and GEE-MD for various combinations of HTE ${\theta }_{3}$ and OTE ${\theta }_{1}$. For scenarios with ${\theta }_{3}={\text{log}}\left(1.5\right)$, we also report the average of cluster sizes obtained by GEE and GEE-KC as a fourth method. Under scenario of prevalence = 30%, the cluster sizes are taken as multiples of ten intentionally to generate integer number of observations for both X = 0 and X = 1 within each cluster. And for the same reason, only even numbers are taken into account under the scenario of prevalence = 50%

Boldfaced simulated power denotes simulated power falls outside of 95% confidence interval for predicted power

**Table 3:** Required cluster sizes to achieve 80% predicted power to detect interaction effect sizes for a nested exchangeable correlation structure with the number of clusters = 8, ICC = 0.1, and CAC = 0.8

${\theta }_{1}$(OTE)	${\theta }_{3}$(HTE)	Method	Prevalence rate for binary individual covariate, X													
			30%							50%						
			Cluster size	$\Delta$ SE	Naïve SE		Robust SE		$\varphi$	Cluster size	$\Delta$ SE	Naïve SE		Robust SE		$\varphi$
					${\psi }_{0}$	${\varphi }_{0}$	${\psi }_{0}$	${\varphi }_{0}$				${\psi }_{0}$	${\varphi }_{0}$	${\psi }_{0}$	${\varphi }_{0}$	
${\text{log}}\left(1.35\right)$	${\text{log}}\left(1.5\right)$	GEE	120	-0.009	0.047	**0.804**	0.118	0.824	0.829	100	0.001	0.050	0.808	0.105	**0.850**	0.806
		Mean(GEE,GEE-KC)	130	-	0.047	0.833	0.124	**0.866**	0.827	110	-	0.047	0.850	0.092	**0.862**	0.806
		GEE-KC	140	0.010	0.054	**0.862**	0.115	**0.881**	0.824	118	0.008	0.048	**0.851**	0.110	**0.888**	0.805
		GEE-MD	160	0.020	0.045	**0.912**	0.106	**0.916**	0.809	140	0.016	0.038	**0.909**	0.116	**0.909**	0.804
	${\text{log}}\left(2\right)$	GEE	40	-0.014	0.037	0.821	0.093	0.847	0.825	34	-0.012	0.054	**0.784**	0.120	0.824	0.809
		GEE-KC	50	0.005	0.055	**0.886**	0.136	**0.902**	0.845	40	0.008	0.058	**0.859**	0.130	**0.881**	0.807
		GEE-MD	60	0.026	0.037	**0.937**	0.126	**0.941**	0.849	48	0.029	0.041	**0.891**	0.113	**0.914**	0.811
${\text{log}}\left(1.68\right)$	${\text{log}}\left(1.5\right)$	GEE	110	-0.000	0.050	**0.777**	0.125	0.825	0.804	96	-0.002	0.043	0.799	0.116	**0.830**	0.801
		Mean(GEE,GEE-KC)	120	-	0.054	0.814	0.118	**0.866**	0.804	106	-	0.044	0.792	0.125	**0.838**	0.801
		GEE-KC	130	0.005	0.036	**0.842**	0.115	**0.874**	0.803	114	0.004	0.047	**0.831**	0.118	**0.864**	0.801
		GEE-MD	160	0.025	0.050	**0.922**	0.127	**0.938**	0.815	136	0.021	0.043	**0.905**	0.128	**0.899**	0.802
	${\text{log}}\left(2\right)$	GEE	40	-0.007	0.044	0.819	0.124	**0.862**	0.830	34	-0.011	0.048	**0.768**	0.115	0.829	0.817
		GEE-KC	50	0.012	0.041	**0.897**	0.115	**0.914**	0.849	40	0.008	0.049	**0.847**	0.128	**0.886**	0.815
		GEE-MD	60	0.033	0.040	**0.935**	0.118	**0.949**	0.853	46	0.030	0.052	**0.884**	0.122	**0.916**	0.802

Estimated required cluster size was determined to achieve at least 80% predicted power. Bias of the standard error $\Delta$ SE, empirical Type I error ${\psi }_{0}$, simulated power ${\varphi }_{0}$ (GEE fitted using both naïve and robust SE, respectively), and predicted power $\varphi$ were obtained from the GEE power calculator with ICC = 0.1 and CAC = 0.8 using three methods GEE, GEE-KC, and GEE-MD for various combinations of HTE ${\theta }_{3}$ and OTE ${\theta }_{1}$. For scenarios with ${\theta }_{3}={\text{log}}\left(1.5\right)$, we also report the average of cluster sizes obtained by GEE and GEE-KC as a fourth method. Under scenario of prevalence = 30%, the cluster sizes are taken as multiples of ten intentionally to generate integer number of observations for both X = 0 and X = 1 within each cluster. And for the same reason, only even numbers are taken into account under the scenario of prevalence = 50%

Boldfaced simulated power denotes simulated power falls outside of 95% confidence interval for predicted power

Based on Tables 2 and 3, statistical significance is determined by the naïve SE, and GEE-MD consistently over-predicts the cluster sizes while GEE consistently under-predicts the cluster sizes. However, when the statistical significance is determined by the robust SE, all three methods tend to consistently over-predict the cluster sizes due to the widely-acknowledged phenomenon that robust SE (without any bias correction) leads to inflated empirical type-I error [13, 35]. Hence the resulting cluster sizes from GEE and GEE-MD may provide a priori range of cluster sizes such that the SW-CRT design may achieve the target power when the data is analyzed with the naïve SE. However, if the data is analyzed with the robust SE, the resulting cluster sizes from GEE are sufficiently large, in which the empirical type-I error should be the major problem. Finally, testing the simulated power/empirical Type I error obtained from the cluster sizes within this range yields more accurate cluster size selection. Moreover, GEE-KC also tends to over-predict the cluster sizes. Hence it is natural to consider the performance of the average cluster size between the cluster sizes obtained by GEE and GEE-KC particularly for the low HTE scenarios when using naïve SE. We add this as a fourth method and report the empirical type-I error and power in Tables 2 and 3 as well. The corresponding predicted power is also directly calculated as the average of predicted powers obtained by GEE and GEE-KC. In Supplementary materials, we show the tradeoff between the number of clusters and cluster size and impact of ICC and CAC on simulated power (Table S1A, Table S1B, and Table S1C). In general, when we have more clusters per step, simulated power increases and when we have more observations per step, the increase in simulated power is slightly bigger than that of fewer observations per step. When ICC and/or CAC decrease, simulated power decreases.

We also perform the same simulation study by replacing the required cluster sizes and predicted powers based on (2) by their analogues based on *t*-distribution with degrees of freedom equal to 4 (we have 8 clusters and 4 cluster level parameters ${\theta }_{0},{\theta }_{1},{\theta }_{2},{\theta }_{3}$, hence we choose the degrees of freedom 4 = 8–4 [36]). In Table S2A and B, the GEE method provides sufficiently large power compared with the corresponding predicted powers when the prevalence is 30%. For prevalence 50%, GEE method shows similar results as the scenarios based on normal Wald test. However, GEE-KC and GEE-MD tend to generate even larger power compared to the corresponding scenarios based on normal Wald test. For the empirical type-I error, the model-based standard error for GEE leads to very small results for t-distribution as shown in Web Fig. 3 in the Supplementary materials of [13].

### Simulated powers

We show simulated powers for a simple exchangeable correlation structure (Table 4) and a nested exchangeable correlation structure (Table 5). For both correlation structures, simulated powers are close to each other but a nested exchangeable correlation structure case has slightly lower power compared to a simple exchangeable correlation structure case. Among three approaches, GEE has the largest simulated powers and GEE-MD has the smallest simulated power. This is a similar pattern as shown in [17] for the OTE. Given cluster size of 120, GEE has over 80% statistical power. When we have a balanced binary covariate (50%), simulated power increases compared to unbalanced binary covariate (30%) as shown in Tables 4 and 5. When we have a large effect size for HTE, simulated power is over 80% for both small and intermediate effect sizes of OTE.

**Table 4:** Simulated powers for a simple exchangeable correlation structure with the number of clusters = 8, cluster size = 120, and ICC = 0.1

	${\theta }_{1}={\text{log}}\left(1.35\right)$				${\theta }_{1}={\text{log}}\left(1.68\right)$			
	${\theta }_{3}={\text{log}}\left(1.5\right)$		${\theta }_{3}={\text{log}}\left(2\right)$		${\theta }_{3}={\text{log}}\left(1.5\right)$		${\theta }_{3}={\text{log}}\left(2\right)$	
Prevalence rate of binary covariate	$30\mathrm{\%}$	$50\mathrm{\%}$	$30\mathrm{\%}$	$50\mathrm{\%}$	$30\mathrm{\%}$	$50\mathrm{\%}$	$30\mathrm{\%}$	$50\mathrm{\%}$
GEE	**0.834**	**0.876**	**0.999**	**1.000**	**0.839**	**0.882**	**0.999**	**1.000**
GEE-KC	0.769	**0.817**	**0.996**	**0.998**	0.775	**0.824**	**0.996**	**0.999**
GEE-MD	0.697	0.748	**0.989**	**0.995**	0.702	0.756	**0.989**	**0.995**

**Table 5:** Simulated powers for a nested exchangeable correlation structure with the number of clusters = 8, cluster size = 120, ICC = 0.1, and CAC = 0.8

	${\theta }_{1}={\text{log}}\left(1.35\right)$				${\theta }_{1}={\text{log}}\left(1.68\right)$			
	${\theta }_{3}={\text{log}}\left(1.5\right)$		${\theta }_{3}={\text{log}}\left(2\right)$		${\theta }_{3}={\text{log}}\left(1.5\right)$		${\theta }_{3}={\text{log}}\left(2\right)$	
Prevalence rate of binary covariate	$30\mathrm{\%}$	$50\mathrm{\%}$	$30\mathrm{\%}$	$50\mathrm{\%}$	$30\mathrm{\%}$	$50\mathrm{\%}$	$30\mathrm{\%}$	$50\mathrm{\%}$
GEE	**0.829**	**0.871**	**0.999**	**1.000**	**0.836**	**0.879**	**0.999**	**1.000**
GEE-KC	0.764	**0.811**	**0.996**	**0.998**	0.771	**0.821**	**0.996**	**0.998**
GEE-MD	0.691	0.743	**0.988**	**0.994**	0.699	0.753	**0.989**	**0.995**

### Simulated powers in the sensitivity analysis

First, we examine the effect of different cluster sizes on the simulated power with the same setups. In Tables 6 and 7, five additional cluster sizes along with cluster size of 120 are considered (20, 40, 60, 80, and 100). With the intermediate effect size of HTE, only cluster sizes of 100 and 120 have more than 80% power using GEE for both correlation structures. With the large effect size of HTE, when cluster size is at least 60, all three approaches have more than 80% power. With cluster size of 40, GEE and GEE-KC have more than 80% power.

**Table 6:** Predicted powers for three effect sizes in combination with different cluster sizes and the number of clusters = 8, prevalence rate = 50%, OTE = log(1.68) for a simple exchangeable correlation structure

Magnitude of HTE with I = 8	method	Cluster size (m)					
		20	40	60	80	100	120
${\theta }_{3}={\text{log}}\left(1.5\right)$	GEE	0.252	0.445	0.606	0.729	**0.819**	**0.882**
	GEE-KC	0.220	0.388	0.536	0.657	0.752	**0.824**
	GEE-MD	0.193	0.336	0.469	0.583	0.679	0.756
${\theta }_{3}={\text{log}}\left(2\right)$	GEE	0.595	**0.875**	**0.968**	**0.992**	**0.998**	**1.000**
	GEE-KC	0.526	**0.816**	**0.938**	**0.981**	**0.995**	**0.999**
	GEE-MD	0.459	0.747	**0.895**	**0.960**	**0.985**	**0.995**

**Table 7:** Predicted powers for three effect sizes in combination with different cluster sizes and the number of clusters = 8, prevalence rate = 50%, OTE = log(1.68) for a nested exchangeable correlation structure

Magnitude of HTE with I = 8	method	Cluster size (m)					
		20	40	60	80	100	120
${\theta }_{3}={\text{log}}\left(1.5\right)$	GEE	0.251	0.444	0.604	0.727	**0.816**	**0.879**
	GEE-KC	0.220	0.387	0.534	0.654	0.749	**0.821**
	GEE-MD	0.192	0.336	0.467	0.581	0.676	0.753
${\theta }_{3}={\text{log}}\left(2\right)$	GEE	0.595	**0.874**	**0.967**	**0.992**	**0.998**	**1.000**
	GEE-KC	0.525	**0.815**	**0.937**	**0.981**	**0.994**	**0.998**
	GEE-MD	0.459	0.746	**0.894**	**0.959**	**0.985**	**0.995**

Next, we examine the effect of different number of clusters on the simulated power. In Tables 8, 9, 10, and 11, we use 20 and 40 for the number of clusters, respectively. When we have 20 clusters, we have more than 80% power for all six different cluster sizes with the large effect size of HTE. When we have a cluster size of 60 or more, all three approaches have more than 80% power with the intermediate effect size of HTE (Tables 8 and 9). When we use 40 clusters, we have more than 80% power across all six different cluster sizes except GEE-MD for cluster size of 20 (Tables 10 and 11).

**Table 8:** Predicted powers for three effect sizes in combination with different cluster sizes and the number of clusters = 20, prevalence rate = 50%, OTE = log(1.68) for a simple exchangeable correlation structure

Magnitude of HTE with I = 20	method	m					
		20	40	60	80	100	120
${\theta }_{3}={\text{log}}\left(1.5\right)$	GEE	0.531	**0.821**	**0.941**	**0.982**	**0.995**	**0.999**
	GEE-KC	0.506	0.797	**0.927**	**0.976**	**0.993**	**0.998**
	GEE-MD	0.481	0.771	**0.911**	**0.968**	**0.989**	**0.997**
${\theta }_{3}={\text{log}}\left(2\right)$	GEE	**0.936**	**0.998**	**1.000**	**1.000**	**1.000**	**1.000**
	GEE-KC	**0.921**	**0.997**	**1.000**	**1.000**	**1.000**	**1.000**
	GEE-MD	**0.904**	**0.996**	**1.000**	**1.000**	**1.000**	**1.000**

**Table 9:** Predicted powers for three effect sizes in combination with different cluster sizes and the number of clusters = 20, prevalence rate = 50%, OTE = log(1.68) for a nested correlation structure

Magnitude of HTE with I = 20	method	m					
		20	40	60	80	100	120
${\theta }_{3}={\text{log}}\left(1.5\right)$	GEE	0.530	**0.820**	**0.940**	**0.982**	**0.995**	**0.999**
	GEE-KC	0.505	0.795	**0.926**	**0.975**	**0.992**	**0.998**
	GEE-MD	0.481	0.770	**0.910**	**0.967**	**0.989**	**0.996**
${\theta }_{3}={\text{log}}\left(2\right)$	GEE	**0.935**	**0.998**	**1.000**	**1.000**	**1.000**	**1.000**
	GEE-KC	**0.921**	**0.997**	**1.000**	**1.000**	**1.000**	**1.000**
	GEE-MD	**0.904**	**0.996**	**1.000**	**1.000**	**1.000**	**1.000**

**Table 10:** Predicted powers for three effect sizes in combination with different cluster sizes and the number of clusters = 40, prevalence rate = 50%, OTE = log(1.68) for a simple exchangeable correlation structure

Magnitude of HTE with I = 40	method	m					
		20	40	60	80	100	120
${\theta }_{3}={\text{log}}\left(1.5\right)$	GEE	**0.821**	**0.983**	**0.999**	**1.000**	**1.000**	**1.000**
	GEE-KC	**0.810**	**0.980**	**0.998**	**1.000**	**1.000**	**1.000**
	GEE-MD	0.798	**0.977**	**0.998**	**1.000**	**1.000**	**1.000**
${\theta }_{3}={\text{log}}\left(2\right)$	GEE	**0.998**	**1.000**	**1.000**	**1.000**	**1.000**	**1.000**
	GEE-KC	**0.998**	**1.000**	**1.000**	**1.000**	**1.000**	**1.000**
	GEE-MD	**0.998**	**1.000**	**1.000**	**1.000**	**1.000**	**1.000**

**Table 11:** Predicted powers for three effect sizes in combination with different cluster sizes and the number of clusters = 40, prevalence rate = 50%, OTE = log(1.68) for a nested exchangeable correlation structure

Magnitude of HTE with I = 40	method	m					
		20	40	60	80	100	120
${\theta }_{3}={\text{log}}\left(1.5\right)$	GEE	**0.821**	**0.982**	**0.999**	**1.000**	**1.000**	**1.000**
	GEE-KC	**0.809**	**0.979**	**0.998**	**1.000**	**1.000**	**1.000**
	GEE-MD	0.797	**0.976**	**0.998**	**1.000**	**1.000**	**1.000**
${\theta }_{3}={\text{log}}\left(2\right)$	GEE	**0.998**	**1.000**	**1.000**	**1.000**	**1.000**	**1.000**
	GEE-KC	**0.998**	**1.000**	**1.000**	**1.000**	**1.000**	**1.000**
	GEE-MD	**0.998**	**1.000**	**1.000**	**1.000**	**1.000**	**1.000**

When we compare the impact of increasing cluster size versus increasing the number of clusters, the latter has slightly higher gain in power. For cluster size change from 20 to 40 with 20 clusters, power increases from 53.1% to 82.1% for GEE; 50.6% to 79.7% for GEE-KC; and 48.1% to 77.1% for GEE-MD (Table 6 first two columns). When the number of clusters changes from 20 to 40 with cluster size fixed at 20, power increases from 53.1% to 82.1% for GEE; 50.6% to 81% for GEE-KC; and 48.1% to 79.8% for GEE-MD (Tables 8 and 10; column 1 each).

## Power calculation for the example

Retrospective data for MSHS oncology practices show a racial disparity in who receives the goals of care (GoC) conversations: 35% of White patients get GoC while only 15% of minority patients get GoC. Hence, the primary objective of this SW-CRT trial is to evaluate whether our intervention of a CDSS guided alert system, combined with training in communication and knowledge about the disparity provided to physicians, in the intervention arm results in a higher increase in the proportion of GoC in minority patients as compared to that in White patients. In other words, the main focus is the HTE, the interaction effect of intervention by race. Our hypothesis is that the rate of GoC increases from 15 to 30% (15% increase) in minority patients and from 35 to 40% (5% increase) in non-minority patients. Design parameters for this SW-CRT are shown in Table 12. Based on this setup, statistical powers to detect the HTE were 17.8% for GEE, 15.4% for GEE-KC, and 13.4% for GEE-MD. Given a cluster size of 15 and 80% power, the effect size of HTE should be more than two times larger than the original value of HTE (= 1.96) in Table 12 (4.24 for GEE, 4.7 for GEE-KC, and 5.51 for GEE-MD). Given an HTE effect size of 1.96, bigger cluster sizes are needed (81 for GEE, 90 for GEE-KC, and 117 for GEE-MD).

**Table 12:** Setup for a motivational example

Value for parameter	Reason for determining value
${\theta }_{0}={\text{log}}\left(0.35/0.65\right)$	the true log odds ratio of the outcome in the control group (${W}_{ijk}=0$) with reference group for binary covariate (${X}_{ijk}=0$) for k^th^ individual in i^th^ cluster at j^th^ period. We assume that prevalence of outcome is 15%
${\gamma }_{1}=0, {\gamma }_{2}=0,{\gamma }_{3}=0,{\gamma }_{4}=0,{\gamma }_{5}=0$	No secular trend
${\theta }_{1}={\text{log}}\left(1.24\right)$ ${\theta }_{2}={\text{log}}\left(0.33\right)$ ${\theta }_{3}={\text{log}}\left(1.96\right)$	Assuming minority group is 33%, OTE and main effect of minority status are based on the hypothesis: GoC increases in minority and non-minority patients are from 15 to 30% and from 35 to 40%, respectively
$I=8,J=5,m=15$	Number of clusters, and time steps. Equal sizes for every cluster
$ICC=0.1$; $CAC=0.8$	For a nested exchangeable correlation structure

## Discussion

Detection of interactions between treatment effects and patient or cluster descriptors in SW-CRT is critical for optimizing the healthcare delivery process. In this article, we propose three different approaches for determining sample size and statistical power for the interaction between binary intervention and patient level covariate in SW-CRT. We show through an illustrative example that a much larger sample size is needed for detecting an interaction effect. In this work we focus on cross-sectional SW-CRT and we deal with two generic correlation structures and two bias-correction techniques. Hence, determination of cluster size should be developed for other types of design for SW-CRT such as closed cohort and open cohort. Other types of bias-correction technique are also considered. In reality, the fixed cluster size assumption is too strong, and unequal cluster sizes should be considered in the sample size determination. In this article we consider binary outcomes, while an extension of this work to consider continuous and censored endpoints along with cluster level covariates is of interest for future study. In this article, we focus on the immediate treatment effect model, though we acknowledge there is the possibility of a time-varying treatment effect as proposed in [37]. Further research should be done for examining different patterns of treatment effect between subgroups.

It is worth noting that in Tables 2 and 3, the empirical Type I errors (${\psi }_{0}$) are well-maintained, which deviates from the pattern of inflated empirical Type I error observed by previous studies [13, 36]. By comparing our study design with these previous studies, we found two potential reasons for the empirical Type I errors not to be inflated: 1) the treatment effect of interest (in this study we focus on HTE while the previous studies [13, 36] focus on the OTE, in Web Fig. 3 in the Supplementary materials of [13] the resulting empirical type-I error under scenarios with small number of clusters is slightly inflated above the 95% upper limit of 0.05 even with the model-based SE); 2) the choices of ICC and CAC for the simulation (in [13] the ICC is chosen to represent the small correlations commonly reported in parallel CRTs. In such cases, using model-based SE might be punished by higher level of misspecification of correlation structure while the robust SE fails to converge due to small number of clusters. As a result, both choices are likely to cause the inflation of empirical type-I error). To investigate these two potential reasons, we compute the difference between the empirical variances based on 1,000 simulations and the model-based calculated variances as shown in Table 13, which indicates that the Naïve (model-based) SE estimator underestimates the true SE (consider the empirical SE as true) for all cases as is well-known. However, when estimating OTE ${\theta }_{1}$ the difference $\widetilde{{\mathbb{V}}ar}\left({\widehat{\theta }}_{1,m}\right)-\widehat{{\mathbb{V}}ar}\left({\widehat{\theta }}_{1,m}\right)$ is quite sensitive to the selection of the ICC $\alpha$ and CAC $\rho$; while using the same data-generating procedure to estimate the HTE ${\theta }_{3}$ the difference $\widetilde{{\mathbb{V}}ar}\left({\widehat{\theta }}_{3,m}\right)-\widehat{{\mathbb{V}}ar}\left({\widehat{\theta }}_{3,m}\right)$ seems to be less sensitive to the selection of the CAC $\rho$. In APPENDIX we show the empirical and model-based calculated variances. The effect of CAC $\rho$ on the model-based $\widetilde{{\mathbb{V}}ar}\left({\widehat{\theta }}_{1,m}\right)$ seems to disappear due to replacing ${{\text{N}}}_{i}$ with ${{\text{M}}}_{i}$ in the inversion of matrix. Similar results can be found in [22] where empirical type-I errors are stably located between 0.04 to 0.06 across all scenarios compared with the inflated type-I error from Fig. 2 of [13]. Last but not least, our selection of ${\theta }_{1}={\text{log}}\left(1.68\right)$ and ${\theta }_{2}={\text{log}}\left(1.5\right)$ happens to reduce the difference between $\widehat{{\mathbb{V}}ar}\left({\widehat{\theta }}_{3,m}\right)$ and $\widetilde{{\mathbb{V}}ar}\left({\widehat{\theta }}_{3,m}\right)$ for the cases with $\rho =1$, which leads to even better results of empirical Type I error in Table 10.

**Table 13:** Difference between empirical variance and the model-based variance calculation for OTE ${\theta }_{1}=0$ and HTE ${\theta }_{3}=0$. The number of clusters $I=8$, the total time steps is $J=5$, the cluster size is $m=40$ for each cluster, the prevalence of ${X}_{ijk}$ is 50% when estimating HTE, ${\gamma }_{j}=0.1\left(j-1\right)$ for $j=\mathrm{1,2},\mathrm{3,4},5$, and ${\theta }_{0}={\text{log}}\left(0.15/0.85\right)$

	$\widehat{{\mathbb{V}}ar}\left({\widehat{\theta }}_{1,m}\right)-\widetilde{{\mathbb{V}}ar}\left({\widehat{\theta }}_{1,m}\right)$		$\widehat{{\mathbb{V}}ar}\left({\widehat{\theta }}_{3,m}\right)-\widetilde{{\mathbb{V}}ar}\left({\widehat{\theta }}_{3,m}\right)$		$\widehat{{\mathbb{V}}ar}\left({\widehat{\theta }}_{3,m}\right)-\widetilde{{\mathbb{V}}ar}\left({\widehat{\theta }}_{3,m}\right)$	
Data generating Procedure	${\text{logit}}\left({\mu }_{ijk}\right)={\theta }_{0}+{\gamma }_{j}$		${\text{logit}}\left({\mu }_{ijk}\right)={\theta }_{0}+{\gamma }_{j}$		${\text{logit}}\left({\mu }_{ijk}\right)={\theta }_{0}+{\gamma }_{j}+{\text{log}}\left(1.68\right){W}_{ij}+{\text{log}}\left(1.5\right){X}_{ijk}$	
	$\alpha =0.1$	$\alpha =0.05$	$\alpha =0.1$	$\alpha =0.05$	$\alpha =0.1$	$\alpha =0.05$
$\rho =1$	$0.0052$	$0.0069$	$0.0061$	$0.0067$	$0.0013$	$0.0019$
$\rho =0.5$	$0.0244$	$0.0149$	$0.0087$	$0.0053$	$0.0086$	$0.0067$

The empirical and model-based calculated variances for the OTE ${\theta }_{1}=0$ and the HTE ${\theta }_{3}=0$ were shown in APPENDIX

The empirical Type I errors for all settings in Table 13 are given in Table 14. We observe the inflated Type I errors for cases with $\rho =0.5$ when estimating the OTE. As to the HTE, the empirical Type I errors are well-maintained for all cases. We acknowledge that there might be other factors affecting the difference $\widetilde{{\mathbb{V}}ar}\left({\widehat{\theta }}_{3,m}\right)-\widehat{{\mathbb{V}}ar}\left({\widehat{\theta }}_{3,m}\right)$ as well as the performance of empirical Type I errors, such as the prevalence of the covariate ${X}_{ijk}$ within each cluster. Thus, the situation might be more complicated than our observation from Tables 13 and 14. We believe that further investigations are needed to explore how the difference $\widetilde{{\mathbb{V}}ar}\left({\widehat{\theta }}_{3,m}\right)-\widehat{{\mathbb{V}}ar}\left({\widehat{\theta }}_{3,m}\right)$ might be controlled such that the empirical Type I error can be maintained well.

**Table 14:** Empirical Type I error for OTE ${\theta }_{1}=0$ and HTE ${\theta }_{3}=0$ based on 1,000 simulations using naïve standard error estimator provided by gee function using R. The number of clusters $I=8$, the total time steps is $J=5$, the cluster size is $m=40$ for each cluster, the prevalence of ${X}_{ijk}$ is 50% when estimating HTE, ${\gamma }_{j}=0.1\left(j-1\right)$ for $j=\mathrm{1,2},\mathrm{3,4},5$, and ${\theta }_{0}={\text{log}}\left(0.15/0.85\right)$

	Estimating OTE		Estimating HTE		Estimating HTE	
Data generating Procedure	${\text{logit}}\left({\mu }_{ijk}\right)={\theta }_{0}+{\gamma }_{j}$		${\text{logit}}\left({\mu }_{ijk}\right)={\theta }_{0}+{\gamma }_{j}$		${\text{logit}}\left({\mu }_{ijk}\right)={\theta }_{0}+{\gamma }_{j}+{\text{log}}\left(1.68\right){W}_{ij}+{\text{log}}\left(1.5\right){X}_{ijk}$	
	$\alpha =0.1$	$\alpha =0.05$	$\alpha =0.1$	$\alpha =0.05$	$\alpha =0.1$	$\alpha =0.05$
$\rho =1$	$0.052$	$0.060$	$0.054$	$0.057$	$0.036$	$0.043$
$\rho =0.5$	$0.279$	$0.174$	$0.048$	$0.048$	$0.047$	$0.048$

The empirical and model-based calculated variances for the OTE ${\theta }_{1}=0$ and the HTE ${\theta }_{3}=0$ were shown in APPENDIX

Our work highlights the importance of properly conducting sample size calculations around the interaction of the exposure and patient level covariate in health disparities research. With the need to introduce interventions aimed at closing the racial gap, properly designing and powering these trials is critical in ensuring statistical results are reliable. The previous trials mentioned in the Background section introduce important and promising interventions, but further potential exists to more directly target the interaction effect. In the case of Cox et al.’s trial [9], the ICU web app is targeted to benefit Black and White families with the same effect size. While beneficial, this alone will not reduce the existing baseline gap between the two subgroups. In the case of Ejem et al.’s trial [8], limited information is provided on how to recreate the racial-disparity targeted sample size calculation. Our work aims to provide researchers with a transparent and accessible tool to design their own SW-CRTs around similar interaction effects. We also acknowledge that the field of health disparities research requires a thoughtful, comprehensive, and nuanced approach. While studying the interaction term is important, investigators should not solely rely on this term when making conclusions about disparities. In addition to the presence or absence of a statistically significant interaction, additional factors may exacerbate health disparities, such as the distribution of the exposure and outcome prevalence across subgroups [38]. We also note that the detection and interpretation of the interaction term is scale-dependent. The comparison of changes in the interaction term may be different when interpreted on a relative risk scale versus a risk difference scale. In this paper we focus the interaction on the odds ratio scale [39]. Our work is one step in the comprehensive framework of working to reduce disparities in healthcare.

## Conclusion

We propose three approaches for cluster size determination given the number of clusters. These methods can be also applied for determining the number of clusters given cluster size. GEE has reasonable operating characteristics for determining cluster size in both intermediate and large effect sizes of HTE. Both GEE-KC and GEE-MD provide relatively large sample sizes compared to GEE. R codes used for this manuscript are available in the Supplementary material.

### Supplementary Information

**Additional file 1.** APPENDIX.

**Additional file 2: Table S1A.** Calculated powers for three effect sizes in combination with number of observations=160 and 800 per step, prevalence rate=50%, OTE=log(1.68) for a simple exchangeable correlation structure (ICC=0.1 and CAC=1).** Table S1B.** Calculated powers for three effect sizes in combination with number of observations=160 and 800 per step, prevalence rate=50%, OTE=log(1.68) for a nested exchangeable correlation structure (ICC=0.1 and CAC=0.8).** Table S1C.** Calculated powers for three effect sizes in combination with number of observations=160 and 800 per step, prevalence rate=50%, OTE=log(1.68) for a nested exchangeable correlation structure (ICC=0.05 and CAC=0.4).** Table S2A.** Required cluster sizes (based on ${t}_{4}$-distribution) to achieve 80% predicted power to detect interaction effect sizes for a simple exchangeable correlation structure with the number of clusters=8 and ICC=0.1.** Table S2B.** Required cluster sizes (based on ${t}_{4}$-distribution) to achieve 80% predicted power to detect interaction effect sizes for a nested exchangeable correlation structure with the number of clusters=8, ICC=0.1, and CAC=0.8.

**Additional file 3.** R codes for power calculation and simulation.

## Data Availability

The datasets analyzed in the simulation study were generated from code available in the GitHub repository: https://github.com/DeukwooKwon/HTE-SWCRT.

## Abbreviations

SW-CRT: Stepped-Wedge Cluster-Randomized Trials
GEE: Generalized estimating equations
OTE: Overall treatment effect
HTE: Heterogeneity of treatment effect
KC: Kauermann and Carroll (correction)
MD: Mancl and DeRouen (correction)
