# Supplementary material for: Power calculation for detecting interaction effect in cross-sectional stepped-wedge cluster randomized trials: an important tool for disparity research
Source: BMC Med Res Methodol. 2024 Mar 2;24:57. doi: 10.1186/s12874-024-02162-0 (PMC11323530; doi:10.1186/s12874-024-02162-0)
Supplement: Supplementary file 1 — Additional file 1. APPENDIX. [file 12874_2024_2162_MOESM1_ESM.docx]

APPENDIX

Formulations for the empirical and model-based calculated variances for the OTE $\theta_{1}=0$

$$\tilde{\mathbb{V}ar}\left( \hat{\theta}_{1,m} \right)=\left[ \left( \sum_{i=1}^{I} \mathbf{N}_{i}^{⊺}\Sigma_{i}^{-1}\mathbf{N}_{i} \right)^{-1} \right]_{6,6}, \hat{\mathbb{V}ar}\left( \hat{\theta}_{1,m} \right)=\frac{1}{999}\sum_{b=1}^{1,000} \left( \hat{\theta}_{1,m}^{\left( b \right)}-\bar{\hat{\theta}_{1,m}} \right)^{2}\approx\frac{1}{999}\sum_{b=1}^{1,000} \left( \hat{\theta}_{1,m}^{\left( b \right)} \right)^{2}$$

Formulations for the empirical and model-based calculated variances for the HTE $\theta_{3}=0$

$$\tilde{\mathbb{V}ar}\left( \hat{\theta}_{3,m} \right)=\left[ \left( \sum_{i=1}^{I} \mathbf{M}_{i}^{⊺}\Sigma_{i}^{-1}\mathbf{M}_{i} \right)^{-1} \right]_{8,8}, \hat{\mathbb{V}ar}\left( \hat{\theta}_{3,m} \right)=\frac{1}{999}\sum_{b=1}^{1,000} \left( \hat{\theta}_{3,m}^{\left( b \right)}-\bar{\hat{\theta}_{3,m}} \right)^{2}\approx\frac{1}{999}\sum_{b=1}^{1,000} \left( \hat{\theta}_{3,m}^{\left( b \right)} \right)^{2}$$

where $\hat{\theta}_{k,m}^{\left( b \right)}$ is estimated by GEE using the $b$th simulated sample for $k=1,3$,

$$\bar{\hat{\theta}_{1,m}}=\frac{1}{1,000}\sum_{b=1}^{1,000} \hat{\theta}_{1,m}^{\left( b \right)}\approx0, \bar{\hat{\theta}_{3,m}}=\frac{1}{1,000}\sum_{b=1}^{1,000} \hat{\theta}_{3,m}^{\left( b \right)}\approx0$$

and

$$\mathbf{N}_{i}=\left[ \begin{matrix} \mathbf{N}_{i11} & \cdots& \mathbf{N}_{i1m_{i}} & \cdots& \mathbf{N}_{iJm_{i}} \end{matrix} \right]^{⊺}\in\mathbb{R}^{Jm_{i}\times\left( J+3 \right)}$$

with $\mathbf{N}_{ijk}:=\left[ \begin{matrix} 1 & e_{j}^{⊺} & W_{ij} \end{matrix} \right]^{⊺}\in\mathbb{R}^{J+3}$.
