# Supplementary material for: Power calculation for detecting interaction effect in cross-sectional stepped-wedge cluster randomized trials: an important tool for disparity research
Source: BMC Med Res Methodol. 2024 Mar 2;24:57. doi: 10.1186/s12874-024-02162-0 (PMC11323530; doi:10.1186/s12874-024-02162-0)
Supplement: Supplementary file 2 — Additional file 2: Table S1A. Calculated powers for three effect sizes in combination with number of observations=160 and 800 per step, prevalence rate=50%, OTE=log(1.68) for a simple exchangeable correlation structure (ICC=0.1 and CAC=1). Table S1B. Calculated powers for three effect sizes in combination with number of observations=160 and 800 per step, prevalence rate=50%, OTE=log(1.68) for a nested exchangeable correlation structure (ICC=0.1 and CAC=0.8). Table S1C. Calculated powers for three effect sizes in combination with number of observations=160 and 800 per step, prevalence rate=50%, OTE=log(1.68) for a nested exchangeable correlation structure (ICC=0.05 and CAC=0.4). Table S2A. Required cluster sizes (based on ${t}_{4}$t4-distribution) to achieve 80% predicted power to detect interaction effect sizes for a simple exchangeable correlation structure with the number of clusters=8 and ICC=0.1. Table S2B. Required cluster sizes (based on ${t}_{4}$t4-distribution) to achieve 80% predicted power to detect interaction effect sizes for a nested exchangeable correlation structure with the number of clusters=8, ICC=0.1, and CAC=0.8. [file 12874_2024_2162_MOESM2_ESM.docx]

SUPPLEMENTARY TABLES

Table S1A. Calculated powers for three effect sizes in combination with number of observations=160 and 800 per step, prevalence rate=50%, OTE=log(1.68) for a simple exchangeable correlation structure (ICC=0.1 and CAC=1)

|  |  | Number of observations per step | | | |
| --- | --- | --- | --- | --- | --- |
|  |  | 160 | | 800 | |
| Magnitude of HTE | method | I=8, m=20 | I=40, m=4 | I=8, m=100 | I=40, m=20 |
| $\theta_{3}=log\left( 1.5 \right)$ | GEE | 0.252 | 0.252 | **0.819** | **0.821** |
|  | GEE-KC | 0.220 | 0.246 | 0.752 | **0.810** |
|  | GEE-MD | 0.193 | 0.240 | 0.679 | 0.798 |
| $\theta_{3}=log\left( 2 \right)$ | GEE | 0.595 | 0.596 | **0.998** | **0.998** |
|  | GEE-KC | 0.526 | 0.583 | **0.995** | **0.998** |
|  | GEE-MD | 0.459 | 0.570 | **0.985** | **0.998** |

Table S1B. Calculated powers for three effect sizes in combination with number of observations=160 and 800 per step, prevalence rate=50%, OTE=log(1.68) for a nested exchangeable correlation structure (ICC=0.1 and CAC=0.8)

|  |  | Number of observations per step | | | |
| --- | --- | --- | --- | --- | --- |
|  |  | 160 | | 800 | |
| Magnitude of HTE | method | I=8, m=20 | I=40, m=4 | I=8, m=100 | I=40, m=20 |
| $\theta_{3}=log\left( 1.5 \right)$ | GEE | 0.251 | 0.252 | **0.816** | **0.821** |
|  | GEE-KC | 0.220 | 0.246 | 0.749 | **0.809** |
|  | GEE-MD | 0.192 | 0.240 | 0.676 | 0.797 |
| $\theta_{3}=log\left( 2 \right)$ | GEE | 0.595 | 0.596 | **0.998** | **0.998** |
|  | GEE-KC | 0.525 | 0.583 | **0.994** | **0.998** |
|  | GEE-MD | 0.459 | 0.570 | **0.985** | **0.998** |

Table S1C. Calculated powers for three effect sizes in combination with number of observations=160 and 800 per step, prevalence rate=50%, OTE=log(1.68) for a nested exchangeable correlation structure (ICC=0.05 and CAC=0.4)

|  |  | Number of observations per step | | | |
| --- | --- | --- | --- | --- | --- |
|  |  | 160 | | 800 | |
| Magnitude of HTE | method | I=8, m=20 | I=40, m=4 | I=8, m=100 | I=40, m=20 |
| $\theta_{3}=log\left( 1.5 \right)$ | GEE | 0.241 | 0.241 | 0.796 | **0.800** |
|  | GEE-KC | 0.211 | 0.235 | 0.727 | 0.788 |
|  | GEE-MD | 0.185 | 0.229 | 0.653 | 0.776 |
| $\theta_{3}=log\left( 2 \right)$ | GEE | 0.572 | 0.573 | **0.997** | **0.998** |
|  | GEE-KC | 0.504 | 0.560 | **0.992** | **0.997** |
|  | GEE-MD | 0.440 | 0.547 | **0.980** | **0.996** |

Table S2A. Required cluster sizes (based on $t_{4}$-distribution) to achieve 80% predicted power to detect interaction effect sizes for a simple exchangeable correlation structure with the number of clusters=8 and ICC=0.1.

| $\theta_{1}$  (OTE) | $\theta_{3}$  (HTE) | Method | Prevalence rate for binary individual covariate, X | | | | | | | |
| --- | --- | --- | --- | --- | --- | --- | --- | --- | --- | --- |
|  |  |  | 30% | | | | 50% | | | |
|  |  |  | Cluster  size | $\psi_{0}$ | $\varphi_{0}$ | $\varphi$ | Cluster  size | $\psi_{0}$ | $\varphi_{0}$ | $\varphi$ |
| $\log\left( 1.35 \right)$ | $\log\left( 1.5 \right)$ | GEE | 200 | 0.003 | 0.839 | 0.816 | 172 | 0.003 | 0.785 | 0.804 |
|  |  | GEE-KC | 240 | 0.005 | **0.878** | 0.804 | 210 | 0.007 | **0.885** | 0.800 |
|  |  | GEE-MD | 270 | 0.004 | **0.919** | 0.800 | 240 | 0.002 | **0.926** | 0.802 |
|  | $\log\left( 2 \right)$ | GEE | 70 | 0.008 | 0.843 | 0.827 | 60 | 0.003 | **0.853** | 0.813 |
|  |  | GEE-KC | 80 | 0.004 | **0.893** | 0.813 | 70 | 0.003 | **0.894** | 0.807 |
|  |  | GEE-MD | 100 | 0.005 | **0.954** | 0.834 | 82 | 0.003 | **0.924** | 0.803 |
| $\log\left( 1.68 \right)$ | $\log\left( 1.5 \right)$ | GEE | 200 | 0.007 | 0.813 | 0.820 | 170 | 0.005 | 0.799 | 0.806 |
|  |  | GEE-KC | 240 | 0.003 | **0.881** | 0.809 | 206 | 0.004 | **0.876** | 0.802 |
|  |  | GEE-MD | 270 | 0.006 | **0.929** | 0.802 | 238 | 0.004 | **0.908** | 0.803 |
|  | $\log\left( 2 \right)$ | GEE | 70 | 0.002 | 0.842 | 0.831 | 58 | 0.006 | 0.804 | 0.807 |
|  |  | GEE-KC | 80 | 0.007 | **0.900** | 0.817 | 68 | 0.004 | **0.873** | 0.804 |
|  |  | GEE-MD | 100 | 0.005 | **0.945** | 0.836 | 82 | 0.004 | **0.915** | 0.809 |

Estimated required cluster size was determined to achieve at least 80% predicted power. Empirical Type I error $\psi_{0}$, simulated power $\varphi_{0}$, and predicted power $\varphi$ were obtained from the GEE power calculator with ICC=0.1 using three methods GEE, GEE-KC, and GEE-MD for various combinations of HTE $\theta_{3}$ and OTE $\theta_{1}$. Under scenario of prevalence=30%, the cluster sizes are taken as multiples of ten intentionally to generate integer number of observations for both X=0 and X=1 within each cluster. And for the same reason, only even numbers are taken into account under the scenario of prevalence=50%.

Boldfaced simulated power denotes simulated power is fallen outside of 95% confidence interval for predicted power.

Table S2B. Required cluster sizes (based on $t_{4}$-distribution) to achieve 80% predicted power to detect interaction effect sizes for a nested exchangeable correlation structure with the number of clusters=8, ICC=0.1, and CAC=0.8.

| $\theta_{1}$  (OTE) | $\theta_{3}$  (HTE) | Method | Prevalence rate for binary individual covariate, X | | | | | | | |
| --- | --- | --- | --- | --- | --- | --- | --- | --- | --- | --- |
|  |  |  | 30% | | | | 50% | | | |
|  |  |  | Cluster  size | $\psi_{0}$ | $\varphi_{0}$ | $\varphi$ | Cluster  size | $\psi_{0}$ | $\varphi_{0}$ | $\varphi$ |
| $\log\left( 1.35 \right)$ | $\log\left( 1.5 \right)$ | GEE | 210 | 0.003 | **0.829** | 0.814 | 180 | 0.004 | 0.811 | 0.801 |
|  |  | GEE-KC | 250 | 0.004 | **0.904** | 0.811 | 216 | 0.004 | **0.913** | 0.800 |
|  |  | GEE-MD | 290 | 0.005 | **0.950** | 0.801 | 258 | 0.007 | **0.949** | 0.803 |
|  | $\log\left( 2 \right)$ | GEE | 70 | 0.004 | 0.838 | 0.820 | 60 | 0.004 | 0.821 | 0.806 |
|  |  | GEE-KC | 80 | 0.005 | **0.888** | 0.806 | 72 | 0.005 | **0.881** | 0.811 |
|  |  | GEE-MD | 100 | 0.004 | **0.956** | 0.824 | 84 | 0.004 | **0.944** | 0.802 |
| $\log\left( 1.68 \right)$ | $\log\left( 1.5 \right)$ | GEE | 200 | 0.004 | **0.832** | 0.806 | 174 | 0.006 | 0.814 | 0.802 |
|  |  | GEE-KC | 240 | 0.003 | **0.896** | 0.808 | 208 | 0.001 | **0.883** | 0.803 |
|  |  | GEE-MD | 290 | 0.002 | **0.933** | 0.813 | 248 | 0.005 | **0.924** | 0.802 |
|  | $\log\left( 2 \right)$ | GEE | 70 | 0.001 | 0.849 | 0.826 | 58 | 0.008 | 0.792 | 0.803 |
|  |  | GEE-KC | 80 | 0.005 | **0.888** | 0.813 | 70 | 0.004 | **0.881** | 0.811 |
|  |  | GEE-MD | 100 | 0.005 | **0.949** | 0.831 | 82 | 0.004 | **0.916** | 0.803 |

Estimated required cluster size was determined to achieve at least 80% predicted power. Empirical Type I error $\psi_{0}$, simulated power $\varphi_{0}$, and predicted power $\varphi$ were obtained from the GEE power calculator with ICC=0.1 and CAC=0.8 using three methods GEE, GEE-KC, and GEE-MD for various combinations of HTE $\theta_{3}$ and OTE $\theta_{1}$. Under scenario of prevalence=30%, the cluster sizes are taken as multiples of ten intentionally to generate integer number of observations for both X=0 and X=1 within each cluster. And for the same reason, only even numbers are taken into account under the scenario of prevalence=50%.

Boldfaced simulated power denotes simulated power is fallen outside of 95% confidence interval for predicted power.
