# Supplementary material for: Power calculation for detecting interaction effect in cross-sectional stepped-wedge cluster randomized trials: an important tool for disparity research
Source: BMC Med Res Methodol. 2024 Mar 2;24:57. doi: 10.1186/s12874-024-02162-0 (PMC11323530; doi:10.1186/s12874-024-02162-0)
Supplement: Supplementary file 3 — Additional file 3. R codes for power calculation and simulation. [file 12874_2024_2162_MOESM3_ESM.docx]

**R CODES FOR POWER CALCULATION AND SIMULATION**

# Power calculation

The predicted power is calculated using the calc.power function. Here are illustrations about how to use the function to obtain the predicted power given the SW-CRT design and model parameters. To obtain the analytic power using the GEE method, one may input in R:

calc.power(m = 40, method = "GEE")

## [1] 0.8739938

To obtain the analytic power using the GEE-KC method, one may input in R:

calc.power(m = 40, method = "GEE-KC")

## [1] 0.8149614

To obtain the analytic power using the GEE-MD method, one may input in R:

calc.power(m = 40, method = "GEE-MD")

## [1] 0.7458535

The function calc.power is given as follows. The inputs m, method, reg.coef, ICC, CAC, prevalences and sig.level can be customized.

library(boot)
library(MultBiplotR)

##
## Attaching package: 'MultBiplotR'

## The following object is masked from 'package:boot':
##
## logit

calc.power <- function(m, # sample size within each cluster and period
 method, # One of "GEE", "GEE-KC", and "GEE-MD"
 reg.coef = c(log(0.15/0.85), # theta_0
 0.1, 0.2, 0.3, 0.4, #gamma's
 log(1.68), # theta_1 (OTE)
 log(1.5), # theta_2
 log(2)), #theta_3 (HTE)
 ICC = 0.1, CAC = 0.8, # alpha and rho
 prevalences = 0.5, # prevalence of X's
 I = 8, # number of clusters
 sig.level = 0.05 # significance level
 ){
 I.matrix <- function(n) diag(rep(1, n)) # Identity matrix of dim n
 J.matrix <- function(n) matrix(1, nrow = n, ncol = n) # Square matrix J_n
 J = length(reg.coef) - 3 #number of periods
 R.list <- lapply(rep(m, I), function(i){
 ICC * CAC * J.matrix(i * J) + ICC * (1 - CAC) * kronecker(I.matrix(J), J.matrix(i)) + (1 - ICC) * I.matrix(i * J)
 }) # Generate the correlation matrices R_i
 S <- J - 1 # must be multiples of I for this setting
 cluster.index <- 1:I
 alloc <- rep(1:S, each = (I/S))
 state.ini <- rep(0, I)
 SW.design <- sapply(1:J, function(s){
 state.ini[which(alloc < s)] <- 1
 return(state.ini)
 }) # Generate the design matrix
 W.list <- lapply(cluster.index, function(i){
 rep(SW.design[i,], each = m)
 })
 e.list <- lapply(cluster.index, function(i){
 rep(0:S, each = m)
 })
 X.list <- lapply(cluster.index, function(i){
 rep(rep(c(1,0), c(floor(m * prevalences), (m - floor(m * prevalences)))), J)
 })
 M.list <- lapply(cluster.index, function(i){
 model.matrix(~ factor(e.list[[i]]) + W.list[[i]] * X.list[[i]])
 }) # Generate matrices M_i
 Asq.list <- lapply(M.list, function(i){
 u0 <- inv.logit(as.numeric(i %*% reg.coef))
 diag(sqrt(u0 * (1 - u0)))
 }) # Generate the square root of matrices A_i
 GEE.list <- lapply(cluster.index, function(i){
 t(M.list[[i]]) %*% Asq.list[[i]] %*% solve(R.list[[i]]) %*% Asq.list[[i]] %*% M.list[[i]]
 })
 FI <- Reduce("+", GEE.list)
 var.GEE <- solve(FI)
 if(method == "GEE"){
 var.interact <- var.GEE[length(reg.coef), length(reg.coef)]
 }
 else if(method == "GEE-KC"){
 Asqinv.list <- lapply(M.list, function(i){
 u0 <- inv.logit(as.numeric(i %*% reg.coef))
 diag(1/sqrt(u0 * (1 - u0)))
 })
 GEE.KC.list <- lapply(cluster.index, function(i){
 grad <- Asq.list[[i]] %*% M.list[[i]]
 H <- Asq.list[[i]] %*% grad %*% var.GEE %*% t(grad) %*% solve(R.list[[i]]) %*% Asqinv.list[[i]]
 F.KC <- matrixsqrtinv(I.matrix(J * m) - H)
 B <- Asq.list[[i]] %*% solve(R.list[[i]]) %*% Asqinv.list[[i]] %*% F.KC
 t(M.list[[i]]) %*% B %*% Asq.list[[i]] %*% R.list[[i]] %*% Asq.list[[i]] %*% t(B) %*% M.list[[i]]
 })
 FI.KC <- Reduce("+", GEE.KC.list)
 var.GEE.KC <- solve(FI) %*% FI.KC %*% solve(FI)
 var.interact <- var.GEE.KC[length(reg.coef), length(reg.coef)]
 }
 else if(method == "GEE-MD"){
 Asqinv.list <- lapply(M.list, function(i){
 u0 <- inv.logit(as.numeric(i %*% reg.coef))
 diag(1/sqrt(u0 * (1 - u0)))
 })
 GEE.MD.list <- lapply(cluster.index, function(i){
 grad <- Asq.list[[i]] %*% M.list[[i]]
 H <- Asq.list[[i]] %*% grad %*% var.GEE %*% t(grad) %*% solve(R.list[[i]]) %*% Asqinv.list[[i]]
 F.MD <- solve(I.matrix(J * m) - H)
 B <- Asq.list[[i]] %*% solve(R.list[[i]]) %*% Asqinv.list[[i]] %*% F.MD
 t(M.list[[i]]) %*% B %*% Asq.list[[i]] %*% R.list[[i]] %*% Asq.list[[i]] %*% t(B) %*% M.list[[i]]
 })
 FI.MD <- Reduce("+", GEE.MD.list)
 var.GEE.MD <- solve(FI) %*% FI.MD %*% solve(FI)
 var.interact <- var.GEE.MD[length(reg.coef), length(reg.coef)]
 }
 else stop("The input method is unavailable.")
 calc.power <- pnorm((reg.coef[length(reg.coef)]
 / sqrt(var.interact) - qnorm(1 - (sig.level / 2)))) + pnorm((-reg.coef[length(reg.coef)]
 / sqrt(var.interact) - qnorm(1 - (sig.level / 2))))
 return(calc.power)
}

# Simulation

Here are the codes for simulating the empirical type-I error and power of an SW-CRT design with 8 clusters and 5 time period. The cluster size for each cluster at each time period is 120 with prevalence 50%. The following code chunk is to set up the design matrix, GEE regression model matrix, and correlation matrices.

library(VGAM)

## Loading required package: stats4

## Loading required package: splines

##
## Attaching package: 'VGAM'

## The following objects are masked from 'package:MultBiplotR':
##
## logit, wine

## The following objects are masked from 'package:boot':
##
## logit, simplex

library(boot)
library(MASS)

##
## Attaching package: 'MASS'

## The following object is masked from 'package:MultBiplotR':
##
## ginv

library(gee)
m <- 120 # Cluster size
n <- 60 # Hence the prevalence is given by n/m
alloc <- c(1, 1, 2, 2, 3, 3, 4, 4)
I <- length(alloc)
cluster.index <- 1:I
S <- length(unique(alloc))
J <- S + 1
X.list <- lapply(cluster.index, function(i){
 rep(rep(c(1,0), c(n, (m - n))), J)
})
X <- unlist(X.list)
state.ini <- rep(0, 8)
(SW.design <- sapply(1:J, function(s){
 state.ini[which(alloc < s)] <- 1
 return(state.ini)
})) # Generate the design matrix

## [,1] [,2] [,3] [,4] [,5]
## [1,] 0 1 1 1 1
## [2,] 0 1 1 1 1
## [3,] 0 0 1 1 1
## [4,] 0 0 1 1 1
## [5,] 0 0 0 1 1
## [6,] 0 0 0 1 1
## [7,] 0 0 0 0 1
## [8,] 0 0 0 0 1

W.list <- lapply(cluster.index, function(i){
 rep(SW.design[i,], each = m)
})
W <- unlist(W.list)
e.list <- lapply(cluster.index, function(i){
 rep(0:S, each = m)
})
e <- unlist(e.list)
M.list <- lapply(cluster.index, function(i){
 model.matrix(~ factor(e.list[[i]]) + W.list[[i]] * X.list[[i]])
})
M <- model.matrix(~ factor(e) + W * X) # The model matrix for GEE
cluster.label <- rep(cluster.index, each = (m * J))
sig.level <- 0.05 # Significance level
ICC <- 0.1
CAC <- 0.8
I.matrix <- function(n) diag(rep(1, n))
J.matrix <- function(n) matrix(1, nrow = n, ncol = n)
R.list <- lapply(rep(m, I), function(i){
 ICC * CAC * J.matrix(i * J) + ICC * (1 - CAC) * kronecker(I.matrix(J), J.matrix(i)) + (1 - ICC) * I.matrix(i * J)
}) # Generate the correlation matrices R_i

## Simulation for the empirical Type I error

Scenarios vary across different levels of OTE and HTE. To simulate the type-I error, we fix that the HTE $\theta_{3}=0$ and would like to check the proportion of significant results out of the 1,000 simulated samples.

Theta0 <- c(log(0.15/0.85), 0.1, 0.2, 0.3, 0.4, log(1.68), log(1.5), 0)
u0.list <- lapply(M.list, function(i){
 inv.logit(as.numeric(i %*% Theta0))
}) # The marginal means u_i
u0 <- inv.logit(M %*% Theta0)
Asq0.list <- lapply(u0.list, function(i){
 diag(sqrt(i * (1 - i)))
}) # The square root inverse of A_i
EYY0.list <- lapply(cluster.index, function(i){
 Asq0.list[[i]] %*% R.list[[i]] %*% Asq0.list[[i]] + u0.list[[i]] %*% t(u0.list[[i]])
}) # The second moment E[YY']
Xi0.list <- lapply(cluster.index, function(i){
 outer(X = 1:length(u0.list[[i]]), Y = 1:length(u0.list[[i]]), FUN = Vectorize(function(j,k){
 if (k == j) {return(1)} else{
 x <- u0.list[[i]][j]
 y <- u0.list[[i]][k]
 a <- EYY0.list[[i]][j,k]
 f <- function(r) pbinormcop(x, y, rho = r) - a
 xi <- uniroot(f, interval = c(-0.99999,0.99999))
 return(xi$root)
 }
 }))
})
Xi0.list <- lapply(cluster.index, function(i){
 Eig.original <- eigen(Xi0.list[[i]])
 Eig.new <- ifelse(Eig.original$values < 0, 0, Eig.original$values)
 newMat <- Eig.original$vectors %*% diag(Eig.new) %*% t(Eig.original$vectors)
 newMat <- newMat/sqrt(diag(newMat) %*% t(diag(newMat)))
 return(newMat)
}) # In case any Xi0 matrix is not positively definite, this code chunk will fix it.
n.sims <- 1000 # Number of simulated samples
set.seed(12345678) # Set the seed
simu.sig0 <- replicate(n.sims, {
 output0 <- tryCatch({ # Sometimes the generated sample is singular such that gee function will report an error. The function tryCatch here is to skip the singular samples and generate a new one.
 Y0.list <- lapply(cluster.index, function(i){
 mvrnorm(n = 1, mu = rep(0, length(u0.list[[i]])), Sigma = Xi0.list[[i]]) < qnorm(u0.list[[i]])
 })
 Y0 <- unlist(Y0.list)
 fit0 <- gee(Y0 ~ factor(e) + W * X, id = cluster.label, family = "binomial"(link = "logit"), corstr = "exchangeable", maxiter = 50, tol = 1e-4)
 summary(fit0)
 }, error=function(err){
 Y0.list <- lapply(cluster.index, function(i){
 mvrnorm(n = 1, mu = rep(0, length(u0.list[[i]])), Sigma = Xi0.list[[i]]) < qnorm(u0.list[[i]])
 })
 Y0 <- unlist(Y0.list)
 fit0 <- gee(Y0 ~ factor(e) + W * X, id = cluster.label, family = "binomial"(link = "logit"), corstr = "exchangeable", maxiter = 50, tol = 1e-4)
 return(summary(fit0))
 })
 1 - pnorm(abs(output0$coefficients["W:X", 3])) < (sig.level / 2) # If significant then return "TRUE"
})
mean(simu.sig0)

## Simulation for the simulated power

To simulate the simulated power, we consider different levels of the HTE. Below are codes for the simulation under the scenario with HTE $\theta_{3}=log\left( 1.5 \right)$. The proportion of significant results out of the 1,000 simulated samples is of our interest.

Theta <- c(log(0.15/0.85), 0.1, 0.2, 0.3, 0.4, log(1.68), log(1.5), log(1.5))
u.list <- lapply(M.list, function(i){
 inv.logit(as.numeric(i %*% Theta))
}) # The marginal means u_i
u <- inv.logit(M %*% Theta)
Asq.list <- lapply(u.list, function(i){
 diag(sqrt(i * (1 - i)))
}) # The square root inverse of A_i
EYY.list <- lapply(cluster.index, function(i){
 Asq.list[[i]] %*% R.list[[i]] %*% Asq.list[[i]] + u.list[[i]] %*% t(u.list[[i]])
}) # The second moment E[YY']
Xi.list <- lapply(cluster.index, function(i){
 outer(X = 1:length(u.list[[i]]), Y = 1:length(u.list[[i]]), FUN = Vectorize(function(j,k){
 if (k == j) {return(1)} else{
 x <- u.list[[i]][j]
 y <- u.list[[i]][k]
 a <- EYY.list[[i]][j,k]
 f <- function(r) pbinormcop(x, y, rho = r) - a
 xi <- uniroot(f, interval = c(-0.99999,0.99999))
 return(xi$root)
 }
 }))
})
Xi.list <- lapply(cluster.index, function(i){
 Eig.original <- eigen(Xi.list[[i]])
 Eig.new <- ifelse(Eig.original$values < 0, 0, Eig.original$values)
 newMat <- Eig.original$vectors %*% diag(Eig.new) %*% t(Eig.original$vectors)
 newMat <- newMat/sqrt(diag(newMat) %*% t(diag(newMat)))
 return(newMat)
}) # In case any Xi matrix is not positively definite, this code chunk will fix it.
n.sims <- 1000 # Number of simulated samples
set.seed(12345678) # Set the seed
simu.sig <- replicate(n.sims, {
 output <- tryCatch({ # Sometimes the generated sample is singular such that gee function will report an error. The function tryCatch here is to skip the singular samples and generate a new one.
 Y.list <- lapply(cluster.index, function(i){
 mvrnorm(n = 1, mu = rep(0, length(u0.list[[i]])), Sigma = Xi.list[[i]]) < qnorm(u.list[[i]])
 })
 Y <- unlist(Y.list)
 fit <- gee(Y ~ factor(e) + W * X, id = cluster.label, family = "binomial"(link = "logit"), corstr = "exchangeable", maxiter = 50, tol = 1e-4)
 summary(fit)
 }, error=function(err){
 Y.list <- lapply(cluster.index, function(i){
 mvrnorm(n = 1, mu = rep(0, length(u0.list[[i]])), Sigma = Xi.list[[i]]) < qnorm(u.list[[i]])
 })
 Y <- unlist(Y.list)
 fit <- gee(Y ~ factor(e) + W * X, id = cluster.label, family = "binomial"(link = "logit"), corstr = "exchangeable", maxiter = 50, tol = 1e-4)
 return(summary(fit))
 })
 1 - pnorm(abs(output$coefficients["W:X", 3])) < (sig.level / 2) # If significant then return "TRUE"
})
mean(simu.sig)
